# Metabolic Regulation of Copper Toxicity during Marine Mussel Embryogenesis

**DOI:** 10.3390/metabo13070838

**Published:** 2023-07-11

**Authors:** Tim Young, Samantha L. Gale, Norman L. C. Ragg, Sylvia G. Sander, David J. Burritt, Billy Benedict, Dung V. Le, Silas G. Villas-Bôas, Andrea C. Alfaro

**Affiliations:** 1 Aquaculture Biotechnology Research Group, Department of Environmental Science, School of Science, Auckland University of Technology, Auckland 1010, New Zealand; 2 Centre for Biomedical and Chemical Sciences, School of Science, Auckland University of Technology, Auckland 1010, New Zealand; 3 School of Biological Sciences, University of Auckland, Private Bag 92019, Auckland 1010, New Zealand; 4 Cawthron Institute, Nelson 7010, New Zealand; 5 Department of Chemistry, University of Otago, P.O. Box 56, Dunedin 9010, New Zealand; 6 Marine Mineral Resources Group, Research Division 4: Dynamics of the Ocean Floor, Magmatic and Hydrothermal Systems, GEOMAR Helmholtz Centre for Ocean Research Kiel, Wischhofstr. 1-3, 24148 Kiel, Germany; 7 Department of Botany, University of Otago, 464 Great King St, Dunedin 9016, New Zealand; 8 Faculty of Fisheries, Vietnam National University of Agriculture, Hanoi 000084, Vietnam

**Keywords:** metabolomics, ecotoxicology, copper speciation, aquaculture, shellfish, mollusc physiology, larval development, embryology, invertebrate biology, oxidative stress

## Abstract

The development of new tools for assessing the health of cultured shellfish larvae is crucial for aquaculture industries to develop and refine hatchery methodologies. We established a large-volume ecotoxicology/health stressor trial, exposing mussel (*Perna canaliculus*) embryos to copper in the presence of ethylenediaminetetraacetic acid (EDTA). GC/MS-based metabolomics was applied to identify potential biomarkers for monitoring embryonic/larval health and to characterise mechanisms of metal toxicity. Cellular viability, developmental abnormalities, larval behaviour, mortality, and a targeted analysis of proteins involved in the regulation of reactive oxygen species were simultaneously evaluated to provide a complementary framework for interpretative purposes and authenticate the metabolomics data. Trace metal analysis and speciation modelling verified EDTA as an effective copper chelator. Toxicity thresholds for *P*. *canaliculus* were low, with 10% developmental abnormalities in D-stage larvae being recorded upon exposure to 1.10 μg·L^−1^ bioavailable copper for 66 h. Sublethal levels of bioavailable copper (0.04 and 1.10 μg·L^−1^) caused coordinated fluctuations in metabolite profiles, which were dependent on development stage, treatment level, and exposure duration. Larvae appeared to successfully employ various mechanisms involving the biosynthesis of antioxidants and a restructuring of energy-related metabolism to alleviate the toxic effects of copper on cells and developing tissues. These results suggest that regulation of trace metal-induced toxicity is tightly linked with metabolism during the early ontogenic development of marine mussels. Lethal-level bioavailable copper (50.3 μg·L^−1^) caused severe metabolic dysregulation after 3 h of exposure, which worsened with time, substantially delayed embryonic development, induced critical oxidative damage, initiated the apoptotic pathway, and resulted in cell/organism death shortly after 18 h of exposure. Metabolite profiling is a useful approach to (1) assess the health status of marine invertebrate embryos and larvae, (2) detect early warning biomarkers for trace metal contamination, and (3) identify novel regulatory mechanisms of copper-induced toxicity.

## 1. Introduction

Copper is an essential trace element involved in many biological processes and is required for the survival of all living organisms [1]. Cells use copper as a structural element in regulatory proteins, and due to its unique redox potential, copper serves as a cofactor for several enzymes that carry out fundamental functions during embryonic development, mitochondrial respiration, iron metabolism, antioxidant defence, and the synthesis of neurotransmitters and neuropeptides [2,3,4,5,6,7]. However, when intracellular concentrations exceed the capacity of cells to sequester the ion, free copper is extremely cytotoxic. Redox cycling between Cu^2+^ and Cu^+^ can catalyse the production of reactive oxygen species (ROS) [8]. Although ROS themselves are essential for many biological processes at low levels (including embryonic development [9]), they cause oxidative stress and damage when generated in excess via the oxidative attack of lipids, proteins, DNA, and other cellular components [10]. Thus, to ensure that metal availability is in accordance with physiological needs, it is crucial that all organisms have mechanisms in place to detect and regulate intracellular copper levels through a controlled balance of uptake, efflux, and sequestration [11,12,13].

Bivalve molluscs have high capacities to bioaccumulate dissolved and particulate-bound metals from seawater. For this reason, bivalves are routinely used as sentinels for trace metal contamination biomonitoring purposes around the world (e.g., the long-running US National Oceanic and Atmospheric Administration’s ‘Mussel Watch’ Program) [14,15]. Some species are, however, particularly sensitive to copper and are disappearing from coastal regions with high loadings of the metal [16,17]. Dissolved copper levels in oceanic and coastal waters typically range from 0.05–1.7 µg·L^−1^, but due to anthropogenic inputs, they have been detected at levels up to 20 µg·L^−1^ in New Zealand and as high as 40 µg·L^−1^ in Australia [18,19,20]. These values far exceed the Australian and New Zealand Environment and Conservation Council 95% protection trigger value of 1.3 µg·L^−1^, and the EC50 embryotoxicity values reported for numerous marine invertebrate species (e.g., mussels, oysters, and sea urchins) [21,22,23,24,25,26,27].

Contributing sources of copper in New Zealand waters include stormwater drainage, leachates from antifouling paints, and run-off from hard-stand activities (e.g., boat-washing, scraping, and repainting) [19]. For the Auckland region alone, it is estimated that up to 22,000 kg of copper are leached from boat hulls every year, with about half of this quantity coming from boats berthed in marinas [28,29]. Copper contamination of New Zealand’s marine environment has the potential to adversely affect wild populations of marine molluscs (as has already been the case for tin [30]) and could pose a threat to the developing aquaculture industry. 

Shellfish farming provides a lucrative source of revenue for New Zealand, and the sector is currently being targeted for expansion [31]. Greenshell^TM^ mussel (*Perna canaliculus*) aquaculture has particularly received considerable investment for growth through shared government–industry funding [32]. Researchers aim to develop technologies to cultivate mussel embryos and larvae at commercial scale to provide farms with high-quality hatchery-reared juveniles for grow-out. This would substantially reduce reliance on wild-sourced stock and provide an excellent avenue to advance the country’s selective breeding programme for improved trait enhancements. *P. canaliculus* is one of New Zealand’s most sensitive endemic bivalves to heavy metal contamination [33], and a number of knowledge gaps exist regarding the toxicity thresholds of certain metals, such as copper, and their toxicological mechanisms in different life history stages (i.e., embryos, larvae, spats, and adults).

Mass larval mortalities in shellfish hatcheries occur at times due to undetermined causes. Potential trace element contamination of bulk seawater used by hatcheries is a general concern for industry, but comprehensive monitoring and source tracking can be very difficult and expensive to implement. The toxicity of metals in seawater is complicated due to speciation and the formation of complexes that alter bioavailable forms. Metal bioavailability is challenging to measure and is strongly impacted by pH, salinity, and the contents of carbonate and organic matter [34]. Such parameters can also be highly dynamic in coastal waters. A common method used by hatcheries worldwide to alleviate concerns of potential trace metal contamination in source seawater is through the use of metal chelators, such as ethylenediaminetetraacetic acid (EDTA). Adding EDTA during embryonic incubation (when shellfish are at their most vulnerable development stage) alters the bioaccumulation of trace metals into larval tissues, ameliorates oxidative stress, and substantially improves the yield/survival of early D-stage larvae [35,36,37]. However, the impact of EDTA additions on hatchery seawater chemistry and metal bioavailability through speciation and complexation is currently unascertained. A better understanding of how embryos and larvae cope with trace metal contamination in the presence of EDTA is also needed, with a focus on biochemical regulatory mechanisms using fine-grained health assessment endpoints to investigate sublethal effects on metabolism and physiology.

In this study, we use passive sampling and a metal speciation modelling approach to measure copper bioavailability in typical EDTA-treated hatchery seawater, with and without excess copper. We also gain industry-relevant information on the toxicity thresholds of copper in early-developing mussels by exposing newly fertilised embryos to copper treatments under simulated hatchery conditions. We further investigate mechanistic aspects of copper toxicity through comprehensive profiling of embryonic/larval metabolites (metabolomics), targeted analyses of enzymes involved in ROS regulation, and evaluation of biomarkers for oxidative damage to lipids, proteins, and DNA.

## 2. Materials and Methods

### 2.1. Experimental Design Summary

Fertilised mussel embryos were exposed to various concentrations of copper in the presence of EDTA during a large-volume hatchery trial at the Cawthron Institute Aquaculture Park (CAP), Nelson, New Zealand. Approximately 150 million embryos were evenly distributed among 15 × 170 L conical plastic tanks containing natural seawater and 4 µM EDTA. The addition of EDTA to the embryonic incubation seawater is current standard commercial practice for the first 48 h of development and is important for the successful development of *P. canaliculus* embryos [36]. Volumes of a copper sulphate stock solution were added to the relevant tanks to provide four nominal levels of total copper (0, 100, 200, 300 µg·L^−1^), with three tank replicates per treatment level. Samples of embryos/larvae were taken recurrently from each tank over a period of 72 h for assessments of cell viability and mortality, development rate, structural abnormalities, swimming behaviour, and metabolite profiling. A comprehensive compositional analysis of the source seawater used in the experiment was conducted through a variety of standard analytical methods. Due to the complexities of incorporating EDTA into the experimental design, analysis of copper speciation during the trial was essential and thus determined via diffusive gradients in thin films (DGTs), passive samplers, inductively coupled plasma mass spectrometry, and a metal speciation modelling approach.

### 2.2. Chemicals and Seawater Preparation

Unless otherwise stated, all reagents were obtained from Sigma-Aldrich Pty Ltd. (Sydney, Australia). Stock solutions of CuSO_4_ (1000 mg·L^−1^ dissolved in Milli-QTM H_2_O; Quantum R Tex cartridge with Biopack polisher) and EDTA disodium salt (5 mM molecular grade [Scharlau: Chemie, Spain] dissolved in Milli-Q H_2_O, and buffered to pH 8.0 with 5 M NaOH) were prepared using acid washed glassware and stored at 4 °C three days prior to use. Embryo incubation tanks were filled with 154.8 L of 16 °C seawater 24 h before the start of the experiment. This seawater had been pumped from the Tasman Bay (Nelson) and filtered to 40 μm (Arkal Filtration Systems CS Ltd.: Jordan, Israel); stored in 25,000 L tanks; pumped sequentially through three in-line filters: 25 μm, 5 μm, and 1 μm; foam fractionated (PPS4 Foam Fractionator–Aquasonic: NSW, Australia); temperature mixed (achieved by drawing from a 10,000 L storage tank maintained at the target temperature via an external loop passing through a titanium plate heat exchanger); and end-of-line 1 µm cartridge filtered (model E1PP10-FG–Filterpure: Auckland, New Zealand).

Once all tanks were filled with seawater, 132 mL of stock EDTA solution was added to each tank (based on prior optimisation trials at CAP; unpublished data) to provide final EDTA concentrations of 4 µM, and gentle aeration was provided (500–1000 mL min^−1^). At the same time, 15 × 15 L fertilisation tanks were filled with 10.2 L of similarly treated seawater, and 12 mL of stock EDTA was added to each tank. The bulk seawater and EDTA in all tanks were given 24 h to mix and become thermally stable at room temperature (17 °C). Two hours prior to adding the fertilised mussel embryos, aliquots of the CuSO_4_ stock solution were administered to the relevant embryo incubation tanks in order to give the systems time to equilibrate/stabilise (i.e., mixtures of dissolved organic matter, inorganic compounds, EDTA-metal complexes, and various other Cu species).

### 2.3. Broodstock Collection and Spawning

Mussel (*P. canaliculus*) broodstock (~300 individuals) were collected 24 h prior to spawning from suspended rope culture in Admiralty Bay (Marlborough Sounds, Nelson, New Zealand) and transported to CAP. Mussels were biosecurity treated according to CAP protocols (i.e., gently scrubbed clean, induced to close with a freshwater dip, and then treated with 0.4% [*v*/*v*] sodium hypochlorite in freshwater for 2–5 min), briefly rehydrated, and then maintained dry overnight under a damp cloth (~16 °C) ready for spawning the next day. Spawning was induced via thermal cycling, and gametes from 50 female and 50 male mussels were collected, pooled by sex, and enumerated via microscopy. Established protocols for broodstock handling, spawning, and fertilisation were employed as previously described in our other works [36,38,39], and citations therein.

### 2.4. Fertilisation and Tank Incubation

Approximately 10.2 million oocytes were added to each of the 15 × 15 L fertilisation tanks containing 10.2 L of 24 h aged seawater incorporating 4 µM EDTA. The sperm pool was diluted with FSW and aliquoted into each tank to provide a ratio of 500 sperm per oocyte. After 30 min, successful fertilisations were confirmed by the presence of polar bodies, and the total contents of each fertilisation tank were transferred into one of the incubation tanks (providing a total incubation seawater volume of 165 L). Ambient air temperature, and therefore tank seawater temperature, was maintained at 17 ± 1 °C throughout the trial (routinely validated by spot thermometer checks of the water in the incubation tanks).

### 2.5. Embryo and Larval Sampling

A range of embryonic and larval samples were taken from each of the incubation tanks at 3, 18, 42, and 72 h post-Cu exposure for various microscopical and biochemical analyses. To first determine embryonic/larval densities, triplicate 2 mL samples were taken from each tank via pipette (whilst homogenising the seawater with a large, perforated plunger), transferred to individual wells of BD FalconTM 24-well Tissue Culture Plates (BD Biosciences: NSW, Australia), and enumerated under a microscope. To assess the cell viability of embryos and larval survival, similar 2 mL samples were taken and characterised using a vital stain (neutral red) in accordance with published protocols [40]. To evaluate temporal developmental parameters, 2 mL samples were taken from each tank (while homogenising tank contents), pooled for each treatment level, gently filtered through a 15 µm mesh sieve, transferred to 1.5 mL Eppendorf tubes, and fixed in Davidson’s solution [41] for subsequent microscopy.

To obtain high biomass samples for metabolomic-based and oxidative stress biomarker analyses (ca. 200,000 pooled individuals per sample), organisms were sampled by opening valves at the bottom of the conical tanks (whilst homogenising) and draining a specific volume (calculated from the embryonic/larval densities) into a 15 µm sieve immersed in seawater. Organisms were gently rinsed in FSW to remove debris/biofloc and treatment solutions, concentrated, transferred to 15 mL Falcon^TM^ centrifuge tubes using 3 mL disposable plastic Pasteur pipettes (LabServ–Thermo Fisher: Auckland, New Zealand), and then made up to 14 mL with FSW. Samples were centrifuged at 4× *g* for 30 s, and 6 mL of seawater was removed before being re-homogenised. Then, 1.5 mL aliquots were transferred to a series of BioStor^TM^ 2 mL RNase/DNase-free cryovials (National Scientific Supply: Claremont, CA, USA). Cryovials were centrifuged at 25× *g* for 30 s, excess seawater removed, then immediately snap-frozen in liquid nitrogen and stored at −80 °C until metabolite extraction. Towards the end of the experiment (at 66 hpf [not 72 hpf due to logistical constraints]), samples of D-stage larvae (containing ~50 individuals) were directly taken via pipette from each incubation tank to assess swimming behaviour and conduct in situ determinations of development stage and the presence/absence of structural abnormalities (e.g., shell deformation) by optical microscopy (Olympus: Model CK-X31).

### 2.6. Seawater Chemistry

#### 2.6.1. Bulk Seawater Composition

The composition of bulk seawater prior to EDTA additions was characterised using standard analytical methods, according to APHA (2005), and included the following: total alkalinity (APHA 2520 B), total organic nitrogen (APHA 4500-Norg D), total ammonical nitrogen (APHA 4500-NH3 F), nitrate and nitrite (APHA 4500-NO_3_^−^ I), dissolved reactive phosphorus (APHA 4500-P G), reactive silica (APHA 4500-SiO_2_ F: modified from flow injection analysis), chloride (APHA 4500 Cl^−^ E: modified from continuous flow analysis), bromide (APHA 4110 B), total organic carbon (APHA 5310 B), and concentrations of up to 32 metals, including total dissolved Cu via ICP-MS (APHA 3125 B). pH was monitored in situ using a calomel glass electrode, and salinity was calculated from the total chloride concentration, where: salinity (ppt) = 1.8066 × 10^−3^ × [Cl^−^] (mg·L^−1^). Temperature and pH readings (recorded with both bench-top Mettler Toledo and mini handheld ISFET pH meters [Shindengen model KS701]) were also performed during every seawater collection, every embryonic/larval sampling, and also during the deployment and retrieval of Diffusive Gradients in Thin Film (DGT) passive samplers.

#### 2.6.2. Copper Speciation Analysis

The actual total dissolved Cu concentrations in an incubation experiment can vary significantly from the intended nominal total dissolved Cu concentration due to contamination and adsorption/desorption processes [42]. In order to assess actual vs. nominal total dissolved Cu concentrations and to evaluate potential rates of Cu loss/gain through bio-uptake and/or adhesion to the tank material and/or evaporation, 500 mL of seawater was sampled from every incubation tank 2 h after embryos had been added and also at the end of the trial (72 h later). Trace metal-clean plastic containers (Nalgene, LabServe–Thermo Fisher: Auckland, New Zealand) were used during seawater sampling. Samples were obtained by immersing a clean 15 µm sieve into the tanks and collecting the embryo-free seawater inside. These samples were immediately chilled to 4 °C and dispatched for analysis to the Centre for Trace Element Analysis (CTEA) (University of Otago, New Zealand) within 12 h of collection. Total dissolved Cu concentrations were measured by ICP-MS using an Agilent 7500ce system with an ESI PFA100 micro-concentric nebulizer, following the manufacturer’s recommendations for robust conditions. Calibration was performed with commercial multi-element solutions (Claritas CLMS-2) with In and Sc as reference elements.

DGT passive samplers were also installed for the duration of the experiment to estimate the bioavailable Cu fraction. The devices house a binding gel, diffusive gel, and membrane filter, and passively accumulate labile species from solution while deployed in situ. Labile species are assimilated by the binding gel in a predictable, rate-controlled manner. After recovery of the DGT samplers, the gel layers are leached in acid and analysed by ICP-MS, which allows the estimation of labile copper [43]. Duplicate DGTs were randomly deployed into one incubation tank for every treatment level, two hours after embryo additions. DGTs were placed adjacent to the air flow to promote the movement of seawater across the devices, and care was taken not to contaminate the membrane filters. At the end of the experiment, DGTs were carefully recovered, packaged at 4 °C, and dispatched to CTEA for ICP-MS analysis.

The DGT-labile metal concentration was calculated according to [43]. The bioavailable Cu concentration is assumed to be the labile fraction of dissolved Cu. Other Cu speciation was modelled using the Visual MINTEQ (v 3.0) program [44]. While the inorganic-bound Cu speciation is defined by the bulk seawater composition, EDTA and DOC at the concentration levels quantified in the samples were used to estimate the free Cu concentration. A fixed humic acid to fulvic acid ratio of 1:9 was used to estimate the humic-bound Cu [45].

### 2.7. Metabolite Analysis

#### 2.7.1. Sample Preparation

Metabolites were extracted using a cold methanol-water method [46], with minor modifications for marine invertebrate embryo and larval tissues. The cryovials containing the frozen larvae were placed on dry ice, and 1000 µL of a cold (–20 °C) 1:1 MeOH:H_2_O solution was added (MeOH [Merck: Darmstadt, Germany]; Milli-Q filtered H_2_O), followed by 20 µL of internal standard (10 mM L-alanine-2,3,3,3-d4 [Sigma-Aldrich: St. Louis, MO, USA]). Samples were partially thawed and vortexed for 1 min, then placed through a series of three freeze–thaw cycles with vortexing in between. Samples were cold-centrifuged (2–4 °C) at 20,800× *g* for 10 min. Supernatants were transferred to 15 mL Falcon tubes and cold-stored on dry ice. A second extraction was performed on the leftover pellets by adding 800 µL of cold 4:1 MeOH:H_2_O, followed by two further freeze–thaw cycles. Samples were centrifuged as previously described, and the paired supernatants were combined from both extractions. The remaining biological materials were re-frozen and stored at −80 °C for subsequent protein analysis. The total volume of the methanol-water extracts was brought up to 6.5 mL with cold Milli-Q water, vortexed briefly to mix, and then frozen at −80 °C. Sample extracts were lyophilised in a 12 L freeze dryer (Labconco Corporation: Kansas city, MO, USA) at −80 °C and 0.03 mbar for 24 h, then derivatised by alkylation.

Methyl chloroformate (MCF) derivatives were prepared to convert amino and non-amino organic acids into volatile carbamates and esters. Lyophilised samples were re-suspended in 400 μL of 1 M sodium hydroxide (Merck: Darmstadt, Germany) and 68 μL of pyridine (Sigma-Aldrich: St. Louis, MO, USA). Mixtures were transferred to KimbleTM silanised borosilicate glass tubes (12 × 75 mm) (ThermoFisher: Auckland, NZ) containing 334 μL of methanol. Then, 20 μL of MCF reagent (Sigma-Aldrich: St. Louis, MO, USA) was added, and samples were vortexed for 30 s. Another 20 μL of MCF was added, followed by vortexing for 30 s. To separate the MCF derivatives from the mixture, 400 μL of chloroform (Merck: Darmstadt, Germany) was added, vortexed for 10 s, followed by the addition of 400 μL of 50 mM sodium bicarbonate (Merck: Darmstadt, Germany) solution and vortexing for a further 10 s. The upper aqueous layer was discarded, and a small amount of anhydrous sodium sulphate (BDH Chemicals: Poole, UK) was added to remove residual H_2_O. The chloroform phase containing the MCF derivatives was transferred to 2 mL amber CG glass vials fitted with inserts (Sigma-Aldrich: St. Louis, MO, USA). A sample blank containing 20 μL of L-alanine-2,3,3,3-d4 was similarly derivatised for QC purposes, along with a separate standard amino acid mix (100 µL, 20 mM [Merk: Darmstadt, Germany]).

#### 2.7.2. GC-MS Analysis

Immediately after derivatisation, the MCF derivatives were injected into a GC-MS system (GC7890 coupled to a MSD5975 [Agilent Technologies], with a quadrupole mass selective detector [EI] operated at 70 eV). The system was equipped with a ZB-1701 GC capillary column (30 m × 250 μm id × 0.15 μm with a 5 m stationary phase [86% dimethylpolysiloxane, 14% cyanopropylphenyl]) (Phenomenex: Torrance, CA, USA). The instrumental setup parameters were conducted according to [47]. Samples (1 µL) were injected in pulsed splitless mode with the injector temperature at 260 °C. The helium gas flow through the GC-column was set at a constant flow of 1 mL min^−1^. The GC-oven temperature was initially held at 45 °C for 2 min, and then raised with a gradient of 9 °C min^−1^ to 180 °C; after 5 min, the temperature was increased to 220 °C at 40 °C min^−1^. After a further 5 min, the temperature was increased at 40 °C min^−1^ to 240 °C and held for 11.5 min. Finally, the temperature was increased at 40 °C min^−1^ until it reached 280 °C where it was held for a further 2 min. The interface temperature was set to 250 °C and the quadrupole temperature was set at 200 °C. The mass spectrometer was operated in scan mode (starting after 6 min; mass range 38–650 a.m.u. at 1.47 scans s^−1^). A derivatised sample blank containing the internal standard, a derivatised standard amino acid mix, a non-derivatised standard alkane mix, and a sample of pure chloroform solvent were also injected and analysed for QC purposes.

#### 2.7.3. Data Pre-Processing and Metabolite Identification

Deconvolution of raw chromatographic data was performed using the Automated Mass Spectral Deconvolution and Identification System (AMDIS v2.66) software (online software distributed by the National Institute of Standards and Technology, USA—http://www.amdis.net/, accessed on 13 February 2021). Metabolite identifications and peak integrations (relative quantification) were conducted using Chemstation Software (Agilent Technologies) and customised R xcms-based scripts [48] to interrogate an in-house mass spectral library of MCF derivatised commercial standards. Analyses were carried out in ‘R’ platform version 2.15.0 (http://www.r-project.org/, accessed on 5 May 2021). Compound identifications were based on matches to both the MS spectrum of the derivatised metabolite and its respective chromatographic retention time. The values are generated from the maximum height of the reference ion for the compound peak. The reference ion used as a measure of abundance for each compound is usually the most abundant fragment and is not the molecular ion.

A Microsoft^®^ Excel file containing peak height data for each metabolite was generated and manually checked for the presence of contaminants (e.g., MCF derivative artefacts and plasticisers). Aberrant records were removed, and the resulting QC-filtered peak intensity values were normalised by the intensity of the internal standard to compensate for potential technical variations (e.g., variable metabolite recoveries).

#### 2.7.4. Statistical Analysis and Data Visualisation

Statistical analysis of the survival data was conducted using SPSS v22.0 statistical software (IBM Corp: Armonk, NY, USA). One-way Analysis of Variance (ANOVA) with Tukey’s Post Hoc Tests was performed separately for each of the three sampling times (18, 42, and 72 h post-Cu exposure) across the treatment levels. Pre-processing and statistical analysis of the metabolite data were conducted using MetaboAnalyst v5.0 [49]. After uploading the .csv files containing the formatted peak intensity tables, the data were autoscaled to meet the distributional requirements for subsequent data analyses.

Lethal and sublethal effects of Cu on the embryonic/larval metabolic profiles were assessed separately due to the occurrence of high mortalities between 18 and 42 h post-exposure of embryos to 50.3 µg·L^−1^ bioavailable Cu. To first investigate the underlying structure of the data, Hierarchical Cluster Analysis (HCA) was performed as an unsupervised classification method to identify inherent sample groupings. Euclidian distance and Ward’s criterion were selected as the measures of distance and aggregation, respectively. Heatmap analysis combined with HCA of all metabolite features identified in the embryonic samples was performed to provide a simple visual summary of the data, and cluster metabolites with similar expression profiles across the samples.

For each exposure duration (3 and 18 h), supervised Projection to Latent Structures Discriminant Analysis (PLS-DA) was performed to construct sample classification models and identify metabolites that contributed most towards group partitioning. Metabolites contributing most to the classification model were identified based on the Variable Importance in Projection (VIP) scores. Metabolites with VIP scores ≥ 1.0 were considered important to the model. The quality of the models was evaluated via the R^2^ and Q^2^ values of the first two latent variables (LVs), which indicate the total variation explained in the data and the cross-validated predicted variation, respectively, using the leave-one-out cross validation (LOOCV) approach.

To identify the metabolite features that were significantly altered by Cu treatment within each timeframe of exposure, Significance Analysis of Microarrays/Metabolites (SAM) and fold-change analyses were performed at 3 and 18 h post-treatment as univariate measures to discriminate the toxicological effects. SAM employed an alpha cutoff of 0.05; global false discovery rates (FDR) are provided.

To assist in the interpretation of the data beyond the investigation of single metabolite variations and examine the metabolomics profiles more comprehensively, secondary bioinformatics procedures were performed to extract various sets of functionally related metabolites involved in the same biochemical pathways. These sets were then analysed together to determine the likelihood of entire pathways being differentially regulated as a response to Cu treatment. All reliably annotated metabolites were mapped into metabolic pathways using the Kyoto Encyclopedia of Genes and Genomes (KEGG) database and assessed via Quantitative Enrichment Analysis (QEA) and Network Topology Analysis (NTA) as described by [50]. QEA and NTA were conducted on the control vs. treatment data for each exposure duration. In order to assess the effect of Cu exposure duration on the differential regulation of the enriched biochemical pathways, the results of the QEA and NTA analyses for each timeframe were extracted and re-plotted together (i.e., overlaid) so that any shifts in the significance of particular metabolic pathways could more easily be visualised and discerned.

To investigate the effects of sublethal Cu exposures, PLS-DA analysis of all metabolomics data across development stages and treatments was first performed. Since some data were missing for particular development stages (due to metabolite levels being zero or below the detection limits), these were replaced using the half minimum approach for ‘not missing at random’ data as recommended by [51]. Mean LV1 and LV2 values (±SE) were plotted on a 2-D score plot to summarise general temporal trends in the developing embryonic/larval mussel metabolome and expose the toxicological effect on metabolism.

As the embryos developed, large phenotypic effects were clearly apparent in the baseline metabolic trajectory. Thus, all data were phenotypically normalised and subsequently re-analysed. The normalisation procedure involved the division of each metabolite peak height by the respective mean control value for each development stage. This process essentially resulted in control sample values being centred around 1.0, and treatment data being expressed as a relative fold change compared to controls for each of the four developmental phenotypes. The produced plot revealed new information that was not discernible in the original plot. To provide a visual summary of the metabolite abundances that were responsible for the positioning of sample groups and the metabolic trajectories in the phenotype-normalised PLS-DA plot, Heatmap analysis with combined HCA of metabolites was performed. For each development stage/exposure duration, mean log_2_ foldchange values relative to control samples were calculated, imported into PermutMatrix (v 1.9.3) software [52], and then clustered using Euclidian distance and Ward’s criterion as measures of distance and aggregation, respectively.

### 2.8. Oxidative Stress/Damage Analysis

#### 2.8.1. Protein, Lipid, and DNA Analysis

Prior to extraction, embryonic and larval samples (each containing approximately 200,000 individuals) were thawed in a 20 °C water bath for 1 min. Total protein was extracted on ice by adding 500 µL of ice-cold enzyme extraction buffer (50 mM potassium phosphate [pH 7.2], containing 0.1 mM Na_2_EDTA, 1 mM EGTA, 125 mM KCl, and 1 mM phenylmethylsulfonyl fluoride) to the samples, followed by mixing with a vortex mixer and homogenising for 1 min using a Mini-Beadbeater-1 and Zirconia/Silica beads (Biospec Products: Bartlesville, OK, USA). The homogenate was centrifuged (Eppendorf 5417R: Eppendorf South Pacific Pty. Ltd.: North Ryde, NSW, Australia) for 15 min at 20,800× *g* and 4 °C. The supernatant, now referred to as the protein extract, was then transferred to a 1.5 mL microcentrifuge tube. The pellet of cell debris was retained for lipid extraction, and the protein extract was subjected to ultrafiltration using Vivaspin^®^ 500 Centrifugal Concentrators with 10,000 MWCO membranes (Sartorius Stedim Biotech Gmbh: Goettingen, Germany) according to the manufacturer’s instructions. The ultrafiltered protein extract was reconstituted with ice-cold 100 mM potassium phosphate (pH 7.2), and protein contents were determined using the Lowry protein assay [53]. Samples were then diluted as required with 100 mM potassium phosphate (pH 7.2) prior to targeted enzyme analyses. Protein carbonyl levels in the reconstituted protein extracts were determined via reaction with 2,4-dinitrophenylhydrazine (DNPH) according to [54] and expressed as nmols of carbonyls per mg protein.

Lipids were extracted from the pellet of cell debris (remaining after protein extraction) by adding 500 µL of methanol-chloroform solution (2:1 *v*/*v*) to the tube. Each sample was left to stand for 1 min, and then 200 µL of chloroform was added and vortex-mixed for 30 s. Deionized water (200 µL) was added, and the extract was mixed again for 30 s. To separate the phases, the resulting homogenate was centrifuged (Eppendorf 5417R) twice for 1 min at 20,800× *g* at ambient temperature. Lipid hydroperoxide levels were determined using the ferric thiocyanate method described by [55], adapted for measurement in a microtitre plate reader. Lipid hydroperoxide levels were determined by measuring the absorbance at 500 nm. A calibration curve with t-butyl hydroperoxide was used, and the lipid hydroperoxide content was calculated as nmol of lipid hydroperoxide per sample. The above assays were carried out using a multilabel counter (Wallac Victor 1420, Perkin Elmer: San Jose, CA, USA) controlled by a PC and fitted with a temperature control cell (set to 25 °C) and an auto-dispenser. The data were acquired and processed using the WorkOut 2.0 software package (Perkin Elmer).

DNA was extracted using an ISOLATE II Genomic DNA Kit (Bioline: N.S.W, Australia), following the manufacturers’ instructions for standard samples, but with minor modifications. After addition of the pre-lysis buffer, samples were homogenised for 1 min using a Mini-Beadbeater-96 and Zirconia/Silica beads (Biospec Products). In addition, the pre-lysis was supplemented with 5 mM deferoxamine and 20 mM EDTA, and the lysis buffer was supplemented with 5 mM deferoxamine. All solutions were deoxygenated by gently bubbling with nitrogen gas for 5 min.

Digested DNA samples were analysed using high-performance liquid chromatography (HPLC), followed by UV detection of guanine and coulometric detection of 8-hydroxy-2′-deoxyguanosine (8-OHdG). The procedure was performed using a C18 reverse-phase column (5 mm, 4.6 mm × 250 mm) (JASCO: Tokyo, Japan), an HPLC system (PerkinElmer: Boston, MA, USA), and an electrochemical detector (5100 Coulochem, ESA: Chelmsford, MA, USA). Separation of DNA was achieved using an isocratic mobile phase (50 mM potassium phosphate [pH 5.5] and 10% methanol) at a flow rate of 1 mL min^−1^, with the column maintained at 30 °C. The analytical cell oxidation potentials of the electrochemical detector were set to 150 mV and 350 mV for electrodes 1 and 2, respectively, with the guard cell potential set at 400 mV. Unmodified nucleosides were detected by their absorbance at 260 nm. Peak data were collected and analysed using a DataCenter 4000 (DataworkX: Brisbane, Australia) general-purpose laboratory data interface and Delta 5.0 chromatography data acquisition and analysis software (DataworkX). The retention times for guanine and 8-OHdG were 12 and 17 min, respectively. Pure solutions of 8-OHdG and guanine (Sigma-Aldrich: St Louis, MO, USA) were prepared in HPLC-grade water (Merck: Darmstadt, Germany) and sterilised by passage through 0.22 μm filters (Millipore: Bedford, MA, USA) to be used as standards. The amount of DNA injected onto the column for each sample was estimated using the signal for guanine, and 8-OHdG was quantified by comparison to external standards.

#### 2.8.2. Antioxidant Enzyme Analysis

Total protein was extracted as previously described for protein carbonyl measurements and followed by enzymatic antioxidant assays for superoxide dismutase (SOD: EC 1.15.1.1), catalase (CAT: EC 1.11.1.6), glutathione peroxidase (GPx: EC 1.11.1.9), glutathione reductase (GR: EC 1.8.1.7), and glutathione-S-tranferase (GST: EC 2.5.1.18) activity using a Perkin Elmer Wallac Victor 1420 multilabel counter.

SOD activity was determined using the microplate assay [56], with minor modifications. 50 μL of protein extract or standard (prepared from bovine liver SOD [Sigma-Aldrich] where one unit of SOD corresponded to the amount of enzyme that inhibited the reduction of cytochrome c by 50% in a coupled system with xanthine oxidase at pH 7.8 and 25 °C) was mixed with 125 μL of freshly prepared reaction solution containing PIPES buffer, pH 7.8, 0.4 mM of o-dianisidine, 0.5 mM of diethylenetriaminepentaacetic acid, and 26 μM of riboflavin. Absorbance at 450 nm was measured immediately (t = 0 min), then samples were illuminated with an 18 W fluorescent lamp placed 12 cm above the plate for 30 min and measured again (t = 30 min). A regression analysis was used to prepare a standard line relating SOD activity to the change in absorbance. Superoxide dismutase activities in the extracts, calculated with reference to the standard line, are expressed as units of SOD per mg of total protein.

CAT activity was determined using the chemiluminescent method, adapted for 96-well microplates [57]. 50 μL of extract or standard (purified bovine liver CAT [Sigma-Aldrich] in homogenization buffer) was mixed with 100 μL of 100 mM phosphate buffer (pH 7.0) containing 100 mM of Na_2_EDTA and 50 μL of 1 μM H_2_O_2_. Samples were then incubated at 25 °C for 30 min, after which 50 μL of a solution containing 20 mM luminol and 11.6 U/mL of horseradish peroxidase (Sigma-Aldrich) was injected into each well. Light emission, the intensity of which was proportional to the amount of H_2_O_2_ remaining in the mixture, was measured. A regression analysis was used to prepare a standard line relating standard CAT activities to the intensity of light emission. CAT activities in the extracts were calculated with reference to the standard line and expressed as µmol of H_2_O_2_ consumed per min per mg of total protein.

GPx activity was determined using the spectrophotometric method [58], adapted for a microplate reader. 20 μL of extract or standard was mixed with 170 μL of assay buffer containing 50 mM of Tris-HCl buffer (pH 7.6), 5 mM of Na_2_EDTA, 0.14 mM of NADPH, 1 mM of GSH, and 3 U/mL of wheat germ glutathione reductase (EC 1.6.4.2) (Sigma-Aldrich). The reaction was initiated by the addition of 20 μL of *t*-butyl hydroperoxide to give a final concentration of 0.2 mM. The consumption of nicotinamide adenine dinucleotide phosphate was monitored at 340 nm every 30 s for 3 min, with the plate shaken automatically before each reading. The GPx activities in the extracts were calculated with reference to a standard line constructed with GPx purified from bovine erythrocytes (Sigma-Aldrich) in extraction buffer. The data are expressed as nmol per min per mg of total protein.

GR activity was determined using the method of [59], with minor modifications. Briefly, 50 μL of extract (diluted extract or standard GR from wheat germ [Sigma-Aldrich] in homogenization buffer) was mixed with 150 μL of 100 mM sodium phosphate buffer (pH 7.6) containing 0.1 mM 5,5′-dithiobis(2-nitrobenzoic acid) and 10 μL of 12 mM NADPH. The reaction was initiated by the injection of 10 μL of 3.25 mM oxidised glutathione, and the absorbance was measured at 415 nm every 30 s for 3 min, with the plate shaken automatically before each reading. The rate of absorbance increase per min was calculated, and a calibration curve relating standard GR activities to the change in absorbance was constructed. GR activities in the extracts were calculated and expressed as nmol of oxidised glutathione reduced per min per mg of total protein at pH 7.6 and 25 °C.

GST activity was determined using the photometric 1-chloro-2,4-dinitrobenzene (CDNB) method according to [60], with minor modifications. The absorbance at 340 nm was measured every 30 s for 3 min, with the plate shaken automatically before each reading. The change in absorbance per minute was calculated and converted into nmol of CDNB conjugated to GSH per min per mg of total protein using the extinction coefficient (E340 = 9.6 mM^−1^ cm^−1^) of the resulting S-2,4-dinitrophenylglutathione.

#### 2.8.3. Reduced Glutathione Analysis

Glutathione (GSH) was extracted and identified during an untargeted metabolomics-based analysis. Due to its high relevance in mechanisms of oxidative stress, glutathione data are presented together with other oxidative stress biomarkers that were targeted. To obtain semi-quantitative GSH data, GC/MS-derived peak intensity values were standardised against the L-Alanine-d4 internal standard (i.e., [glutathione peak height/int. std. peak height] x int. std. concentration), assuming a similar response factor of 1.

#### 2.8.4. Analysis of Reactive Oxygen Species

ROS production was measured in vivo using the cell-permeant ROS-detecting fluorescent dye 2′,7′-dichlorodihydrofluorescein diacetate (H_2_-DCFDA) (Invitrogen–Molecular Probes, D399: Eugene, OR, USA). Upon cleavage of the acetate groups by intracellular esterases and oxidation, the non-fluorescent H_2_-DCFDA is converted to the highly fluorescent 2′,7′-dichlorofluorescein (DCF), which can be measured via fluorescence spectroscopy. At 18, 36, and 66 h post-fertilisation (the final sampling time for this analysis was not 72 h due to logistical constraints), approximately 60,000 embryos or larvae from each of the 15 incubation tanks were concentrated on a 15 µm mesh screen, transferred via pipette to a 50 mL FalconTM tube, and made up to 25 mL with FSW. Six 210 µL subsamples (technical replicates) of each larval solution, containing approximately 500 larvae, were then transferred to wells of a 96-well microplate. Blank seawater controls contained 210 µL of FSW. Immediately prior to performing each ROS assay, a pre-frozen stock of the H_2_-DCFDA probe (1 mM in DSMO) was thawed and a 20 × dilution was prepared in FSW. Then, 40 µL of this diluted H_2_-DCFDA working solution was pipetted into all wells, and the plates were incubated in the dark at 19 ± 0.5 °C for 120 min. ROS production was assessed by measuring the DCF fluorescence intensity on a Perkin Elmer microplate reader (Ex/Em: 490/520 nm). Variations in intensity values are reflective of relative differences in ROS production among treatments.

#### 2.8.5. Statistics and Data Presentation

To identify differential expressions/activities of targeted oxidative stress biomarkers in Cu-exposed larvae compared to control organisms, each biomarker was assessed separately via t-test analysis for each development stage and treatment level using SPSS v22.0 (IBM Corporation: Armonk, NY, USA). To facilitate visualisation of the trends on a common scale, the data have been autoscaled and each biomarker presented separately in line plots. A summary of results is also displayed in the form of a table with colour coding, where up/down arrows represent statistically different (*p* < 0.05) biomarker expressions/activities in the Cu-exposed embryos and larvae compared to non-exposed control organisms.

## 3. Results

### 3.1. Seawater Chemistry

The composition of the 1 µm filtered bulk seawater used for the experiment prior to additions of EDTA and CuSO_4_ is presented in Table 1. The salinity and pH (free scale) were 34.3 ppt and 8.1, respectively. After EDTA and CuSO_4_ additions, the actual values of total dissolved Cu were slightly higher than nominally designed, and between-treatment compositional differences in Cu fractionation was verified (Table 2). The bioavailable Cu previously present in the source seawater was almost entirely chelated (99.6%) by the addition of EDTA in the control tanks. EDTA addition also made substantial amounts of Cu biologically unavailable in the treatment tanks. The ability of 4 µM EDTA to complex high levels of toxic Cu species became limiting when the measured total dissolved Cu was between 250 µg·L^−1^ (96% EDTA-bound) and 370 µg·L^−1^ (70% EDTA-bound). Final treatment levels of bioavailable Cu were determined to be 0.04, 1.10, and 50.30 µg·L^−1^. The pH and water temperatures in the incubation tanks did not change significantly throughout the experiment.

### 3.2. Survival and Development

**Figure 1:**
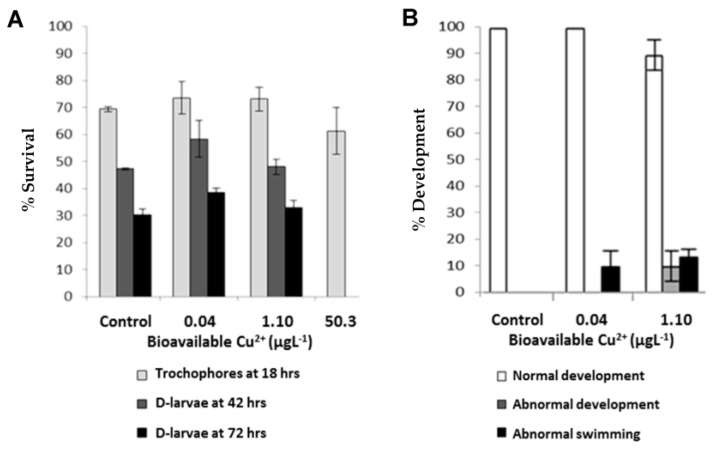
Effects of copper exposure on embryonic/larval survival, larval development, and larval swimming behaviour: (**A**) Percent of organisms surviving at 18, 42, and 72 h post-fertilisation when exposed to sublethal and lethal Cu levels; (**B**) Percent of normally developed D-larvae, abnormally developed D-larvae (very small and/or with shell structure deformities), and incidence of abnormal swimming behaviour (static or slowed movement) after 66 h of exposure to sublethal Cu level, compared to controls. No survivors were found within the 50.3 µg·L^−1^ Cu treatment.

At sublethal bioavailable Cu-exposure levels (0.04 and 1.10 µg·L^−1^), proportions of surviving embryos and larvae at each sampling time (18, 42, and 72 h post-fertilisation [hpf]) were comparable to those surviving in the control tanks (*t*-tests; *p* > 0.05) (Figure 1A). Although exposure of embryos to 50.3 µg·L^−1^ of bioavailable Cu did not compromise survival for the first 18 h significantly, it is likely that they perished soon afterwards as there were no visible indications of intact biological material present during a 36 h post-treatment spot-check. A visual assessment during the mortality assay after 18 h of high Cu exposure revealed that those embryos were also substantially delayed in their development. These comprised mostly of 16 to 32-cell blastulae, some gastrulae, and a few non-swimming trochophores with evidence of abnormal cell differentiation when compared to normally developed and highly mobile trochophore-stage embryos in control tanks. In addition, although cell membrane integrity appeared to be mostly maintained in embryos exposed for 18 h to the high Cu treatment (i.e., demonstrated by the active transport of neutral red across the membrane and permanent incorporation into lysosomes), there were some indications of changes in cell morphology and size (mostly through oncosis, but with some shrinkage also observed), cellular injury and blebbing, and a commencing of loss of cellular adhesion (apparent as cellular dissociation). These observations strongly suggest that the initiation of Cu-induced mechanisms of cellular apoptosis and/or necrosis was occurring in these samples.

Of the surviving organisms towards the end of the experiment, prolonged exposure to Cu resulted in proportions of D-larvae displaying signs of reduced swimming velocity and abnormal motion trajectories (Figure 1B). Approximately 10% of the D-larvae that had been exposed to 1.10 µg·L^−1^ bioavailable Cu also presented developmental abnormalities, such as retarded growth and deformities in shell structure.

### 3.3. Untargeted Metabolomics Analyses

#### 3.3.1. Lethal Exposure Effects

**Figure 2:**
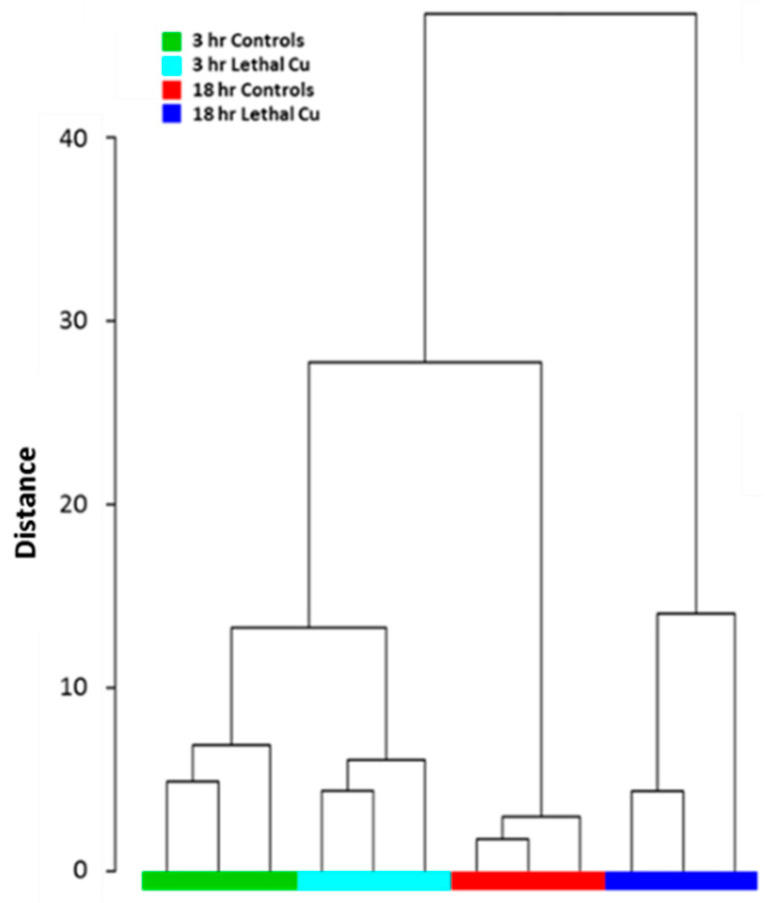
Hierarchical Cluster Analysis (Euclidian distance; Ward’s criterion) of embryo samples (branches) based on global compositional changes in metabolites as a response to 50.3 µg·L^−1^ bioavailable Cu exposure.

Exposure of embryos to 50.3 µg·L^−1^ bioavailable Cu caused extensive metabolic alterations prior to cell death. Hierarchical cluster analysis (HCA) of the 3 h and 18 h Cu-exposed groups and their respective controls revealed that all samples were correctly clustered based on the underlying structure of the metabolite data (Figure 2).

**Figure 3:**
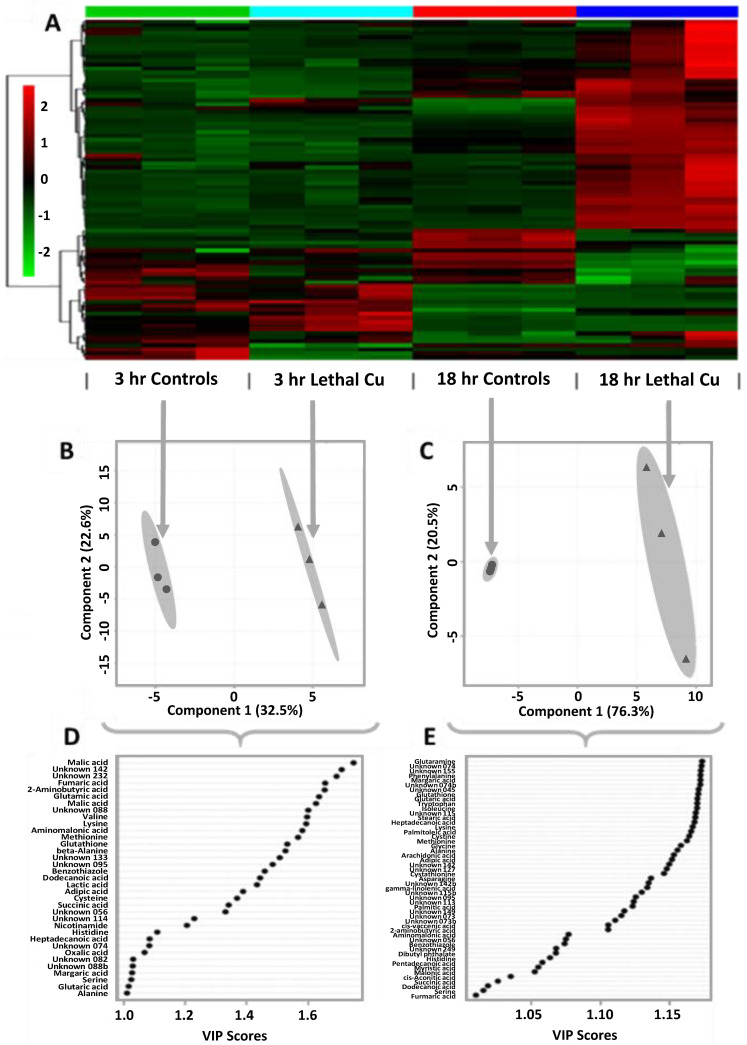
Toxicological effects of high Cu levels (50.3 µg·L^−1^ bioavailable Cu) on the embryonic metabolome after 3 and 18 h of exposure: (**A**) Heatmap of metabolite levels (red > green) with combined HCA of all metabolite features (rows where n = 90); (**B**,**C**) Projection to Latent Structures Discriminate Analysis (PLS-DA) plots of 3 h and 18 h exposed embryos, respectively, compared to their control groups; (**D**,**E**) Variable of Importance (VIP) scores for each of the PLS-DA models.

**Figure 4:**
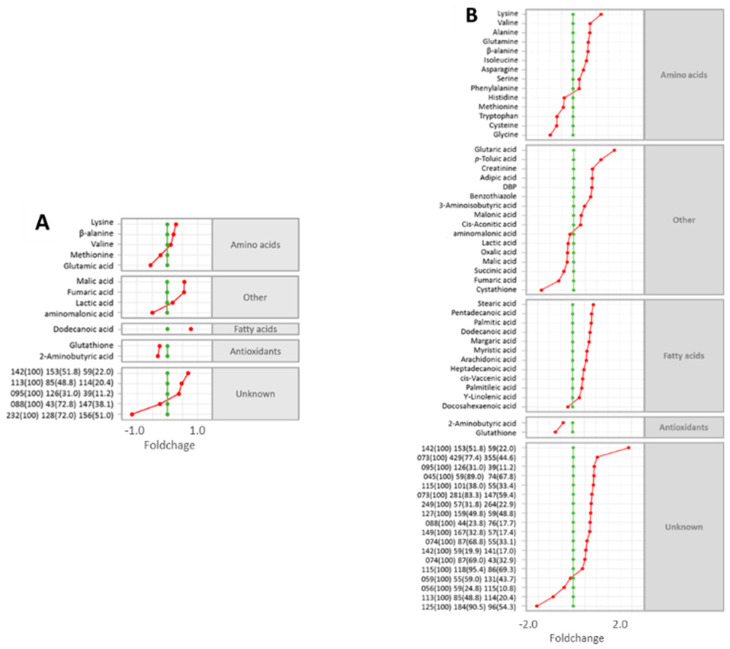
Lists of significantly altered (*p* < 0.05) metabolites detected via SAM analysis in embryos exposed to elevated (50.3 µg·L^−1^ bioavailable) Cu for: (**A**) 3 h (global FDR = 0.11); or (**B**) 18 h (global FDR = 0.01). Red circles (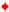) indicate metabolite fold changes in Cu-treated embryos relative to control embryos (green circles; 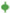) plotted using a log_2_ scale.

Heatmap analysis (Figure 3A) provides a clear visualisation of the global differences/similarities in the metabolite expression profiles that were responsible for the distinct clustering of samples. Separate projection to latent structures discriminant analysis (PLS-DA) models (Figure 3B,C) were constructed for each development stage due to the large influence of the baseline developmental phenotype on the embryonic metabolomes (i.e., 3 h vs. 18 h control samples), and the differential metabolic responses to high Cu levels between these groups. For the 3 h Cu-exposed embryos (Figure 3B), the first latent variable (LV1) accounted for 32.5% of the explained variation between sample groups in the PLS-DA model (R^2^ = 0.99; Q^2^ = 0.61). Analysis of the variable importance in projection (VIP) scores (Figure 3D) revealed that the abundances of 35 features contributed most (i.e., VIP ≥ 1.0) towards sample classification, including metabolites associated with energy production (i.e., succinic, fumaric, and malic acids), lipid metabolism (i.e., free fatty acids), and protein metabolism and osmotic regulation (i.e., free amino acids), among others. Significance analysis of metabolites (SAM) (Figure 4A) identified 17 metabolites that were statistically different between control and treatment groups after 3 h of exposure, with varying directions of expression (positive vs. negative fold changes).

After 18 h of exposure, the effect of elevated Cu on the embryonic metabolome was far more pronounced. PLS-DA analysis of the 18 h Cu-exposed embryos (Figure 3C) resulted in LV1 accounting for 76.3% of the explained variation between groups in the classification model (R^2^ > 0.99; Q^2^ > 0.99). Of the total number of metabolites detected, a substantial proportion (61%) contributed highly (VIP ≥ 1.0) towards the PLS-DA model (Figure 3E). After 18 h of exposure, SAM analysis (Figure 4B) identified 62 metabolites, including 18 unannotated metabolites/features, with abundances that were statistically different between the control and treatment groups.

The majority of metabolites that were differentially expressed after 3 h were also differentially expressed after 18 h (Figure 4). Many of these metabolites also displayed similar foldchange directions. For example, lysine, β-alanine, valine, dodecanoic acid, and an unassigned feature (142(100) 153(51.8)…) were all elevated in the Cu-exposed groups, whereas methionine, aminomalonic acid, glutathione, and 2-aminobutyric acid were similarly reduced. Conversely, a number of metabolites exhibited differential levels that were dependent on the duration of Cu exposure. For example, after 3 h, the abundances of some energy-associated metabolites (malic, fumaric, and lactic acids) were all elevated compared to control embryos; but after a further 15 h of Cu exposure, the expression patterns of these functionally related metabolites fell below control levels.

**Figure 5:**
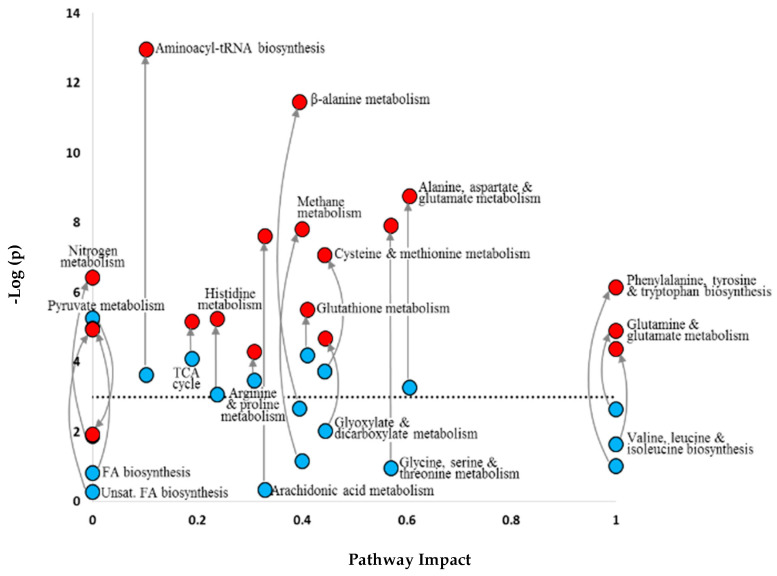
Pathway enrichment and topology analysis of the effects of elevated bioavailable Cu (50.3 µg·L^−1^) on embryos after 3 h (blue circles) and 18 h (red circles) exposure, compared to their respective controls. The *y*-axis represents the −log of the raw *p*-value (i.e., ln[x]) associated with pathway enrichment analysis, and the *x*-axis represents the Pathway Impact (PI) score associated with topology analysis (a measure of metabolite centrality within the pathways). The black dotted line denotes the threshold *p*-value of significance, where pathways below the line (*p* > 0.05) were identified as not being significantly different from those functioning in control embryos. The effects of Cu exposure duration on differentially enriched pathways are seen as vertical shifts in significance.

Quantitative enrichment analysis and network topology analysis of the 3 h and 18 h metabolite datasets (treatment vs. control) exposed some major changes in pathway regulation, which could be compared when overlaid (Figure 5). After 3 h of Cu exposure, eight pathways were identified as being differentially regulated compared to control embryos. These comprise networks associated with energy production, glutathione metabolism, the biosynthesis of aminoacyl-tRNA, and various subsets of amino acid metabolism. After 18 h of Cu exposure, all but one of these pathways (pyruvate metabolism) became significantly disturbed compared to the metabolic baseline controls, as revealed by the pronounced vertical movement of the data. The extended exposure duration also resulted in a further 10 pathways displaying differential regulation compared to control embryos. These included networks associated with extra subsets of amino acid metabolism, methane metabolism, glyoxylate and dicarboxylate metabolism, arachidonic metabolism, and fatty acid biosynthesis.

#### 3.3.2. Sublethal Exposure Effects

**Figure 6:**
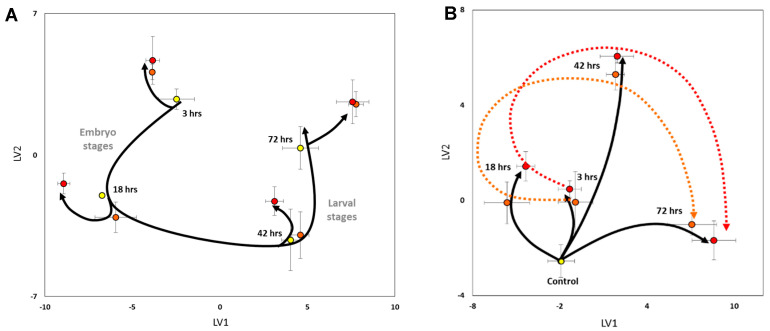
Metabolic trajectory analyses of embryos exposed to sub-lethal Cu^2+^ levels derived from PLS-DA of control embryos (yellow circles) and embryos continuously exposed to 0.04 g·L^−1^ (orange circles) and 1.10 g·L^−1^ (red circles) bioavailable Cu: (**A**) Data are presented as means ± SD of triplicate samples. Black lines/arrows summarise the main trends/trajectories during baseline embryonic development and in response to sublethal Cu exposures for each treatment duration; (**B**) Phenotype-normalised metabolic trajectory analysis derived from PLS-DA of control embryos (yellow circle) and embryos continuously exposed to 0.04 g·L^−1^ (orange circles) and 1.10 g·L^−1^ (red circles) bioavailable Cu. Control data are presented as the mean ± SD of 12 replicates (i.e., triplicate samples for each development stage/exposure duration), whereas treatment data are presented as the mean ± SD of triplicate samples. Black lines/arrows summarise the main metabolic trends/trajectories in embryos/larvae for each exposure duration, and the coloured dashed lines summarise the metabolic trends/trajectories of embryos exposed to each Cu level during the experiment.

Exposure of mussel embryos to sublethal levels of bioavailable Cu (0.04 and 1.10 µg·L^−1^) revealed that even low concentrations had substantial effects on metabolism (Figure 6). These metabolic responses were dependent both on the concentration and duration of Cu exposure. PLS-DA analysis of the sublethal metabolomics data across all samples revealed that there was a clear metabolic trajectory associated with developmental timing (Figure 6A). For each Cu exposure duration, deviations from this baseline trajectory were apparent. The positioning of treated samples within the PLS-DA score plot, relative to their respective controls, indicated that different metabolites were responsible for variations in treatment-induced trajectories along the LV1 and LV2 axes.

Due to the large effect of developmental timing on the embryonic metabolome, the subtleties of some treatment effects were masked, especially when their trajectories aligned with the metabolic path during baseline development (i.e., exposure of embryos to 0.04 µg·L^−1^ bioavailable Cu after 18 and 42 h of exposure). Thus, to remove unimportant components of the global variation contributing towards group partitioning in the model and visually capture the effects of Cu treatments without developmental-induced biases, phenotypic normalisation was applied, and the resulting data were replotted (Figure 6B).

The phenotype-normalised metabolic trajectory plot (Figure 6B) revealed that low and medium bioavailable Cu treatment levels substantially perturbed embryonic and larval metabolic signatures compared to controls. For each exposure duration, the divergent trajectory directions and lengths along the LV1 and LV2 axes further substantiate that the relative abundances of different metabolites are responsible for the observed pattern and provide evidence that the toxic effect of Cu on the embryonic/larval metabolome is time-dependent. Furthermore, there appear to be similar temporal treatment effects between the two Cu treatment levels (Figure 6B, dotted lines), which could not be discerned prior to phenotypic normalisation. This trend is consistently offset between the two Cu treatment levels (with the higher dose being further away from controls than the lower dose), which indicates that higher Cu exposures cause more pronounced disturbances to baseline metabolism than the lower Cu dose, regardless of exposure duration or development stage. However, the positioning of the samples between Cu treatments within each exposure duration is relatively close to one another, indicating that the exposure duration had more of an effect than the copper concentration.

**Figure 7:**
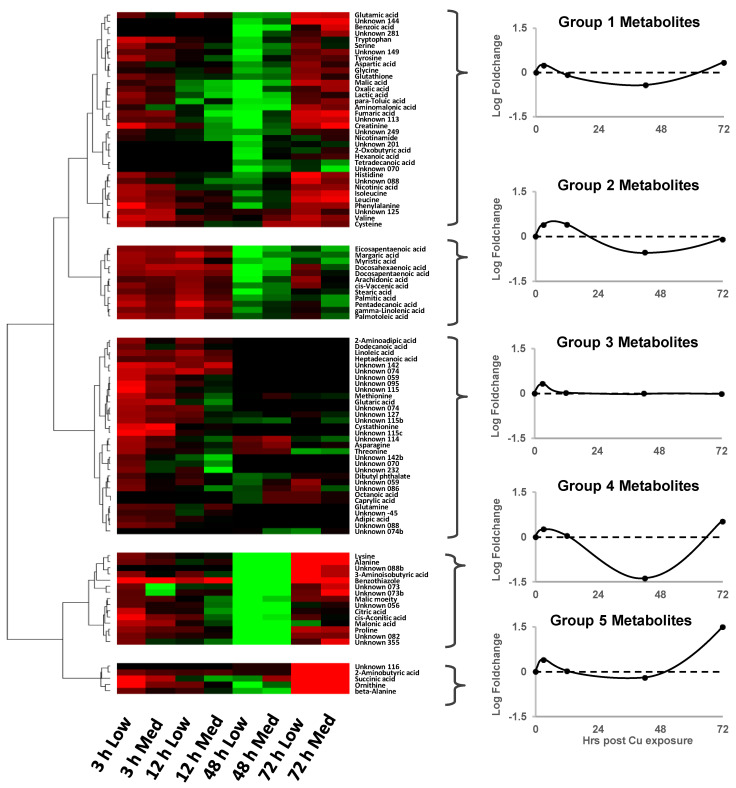
Heatmap and cluster analysis (**left**) of mean metabolite foldchanges in Cu-treated organisms relative to their respective controls, where: Tn = exposure duration (h); Low = 0.04 µg·L^−1^ bioavailable Cu; Med = 1.10 µg·L^−1^ bioavailable Cu. Five general treatment-averaged foldchange trends are also displayed (line plots on the **right**) based on the cluster analysis of metabolites.

After 3 h, most metabolites increased in their relative abundances compared to non-Cu-treated controls (Figure 7). After 18 h, many metabolite levels within the low Cu dose exposure group remained higher than in control embryos, whereas levels of various metabolites in the medium dose exposure group had dropped below control values. After 42 h, the majority of metabolites consistently had negative foldchange values (ca. −2.0) compared to control organisms. These observations suggest that the higher of the two Cu doses initiated the temporal change in metabolite profiles much earlier (after 18 h in trochophore-stage embryos) than the lower treatment dose (apparent after 42 h in D-stage larvae). Continued exposure to sublethal Cu levels resulted in differential metabolite responses after 72 h. The relative abundances of a few metabolites (e.g., succinic acid and 2-aminobutyric acid [Group 5]) increased substantially above control values, many metabolites (e.g., glutamic acid, histidine, and glycine [Group 1]) appeared to return to levels corresponding with those found after 3 h of exposure, and a number of metabolites (e.g., fatty acids [Group 2]) had returned to control levels. A large proportion of metabolites comprising some amino acids and unknown compounds (e.g., asparagine, threonine, and methionine [Group 3]) were minimally impacted. These general trends are summarised in plots of the mean Cu-induced foldchange values for each exposure duration based on the cluster analysis of metabolites (Figure 7 line plots); five broad response patterns were identified.

### 3.4. Targeted Analysis of the ROS Regulatory System

**Figure 8:**
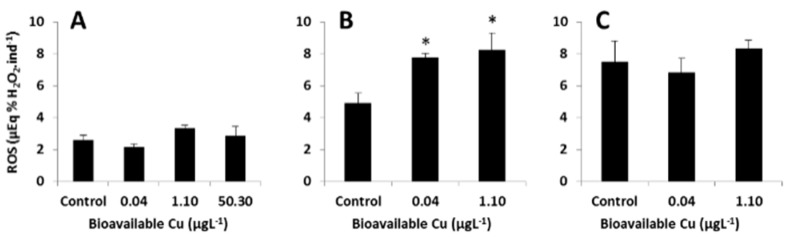
Production of reactive oxygen species (ROS) in embryos and larvae reared under different levels of bioavailable Cu for (**A**) 18 h, (**B**) 36 h, and (**C**) 66 h. ROS levels are expressed as % H_2_O_2_ micro-equivalents per individual. Asterisks denote significant differences compared to controls (ANOVA; Dunnet’s post hoc tests; *p* < 0.05).

Exposure of mussel embryos to copper induced a number of significant changes in the oxidative stress biomarkers assayed. These effects were dependent on the dose and duration of exposure. After 18 h of exposure, levels of embryonic ROS were similar for all treatment groups (Figure 8A). After 36 h of exposure to sub-lethal Cu concentrations, ROS production was significantly elevated (ANOVA; Tukey’s post hoc tests; *p* < 0.05) compared to controls (Figure 8B). A re-balance of the ROS regulatory system was seemingly achieved upon prolonged exposure, with levels resembling the control after 66 h of exposure (Figure 8C).

**Figure 9:**
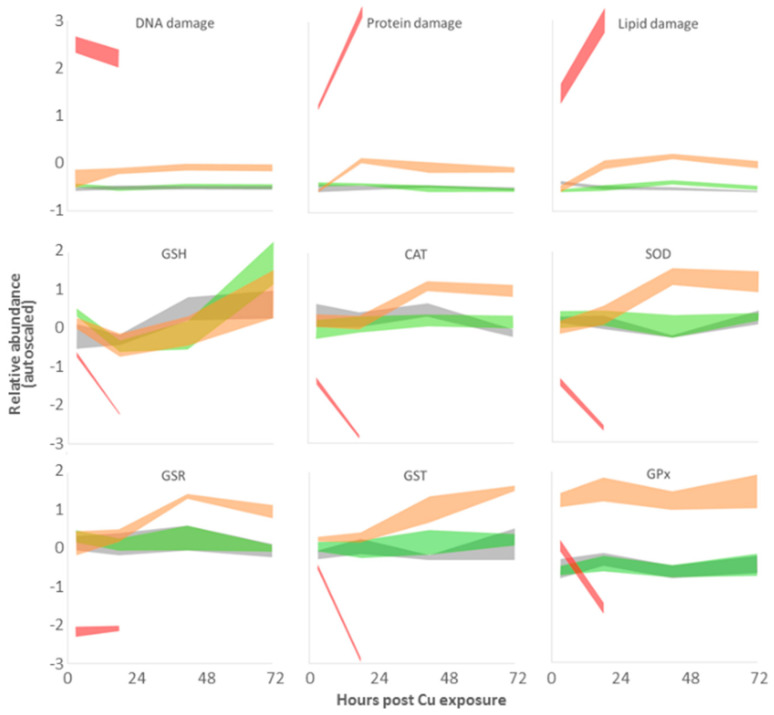
Targeted analysis of oxidative stress biomarkers in mussel embryos incubated in different copper environments. Data are autoscaled, and the widths of coloured lines are bounded by the standard error of the means of triplicate samples of pooled embryos/larvae (n ≈ 200,000), where grey = controls; green = 0.04 µg·L^−1^ bioavailable Cu; orange = 1.10 µg·L^−1^ bioavailable Cu; red = 50.3 µg·L^−1^ bioavailable Cu.

Evidence of oxidative stress responses to copper exposure was revealed in the analysis of enzymes involved in balancing intracellular ROS, and through elevated levels of macromolecular oxidation products (Figure 9; Table 3). Exposure of embryos to low bioavailable Cu (0.04 µg·L^−1^) for 42 h resulted in subtle signs of lipid damage (ca. 13% higher lipid peroxides than control values); with this one exception, macromolecule damage was not significant. This demonstrates that the low-dose treatment did not result in lasting negative effects associated with the characteristic mechanism of heavy metal toxicity via ROS-induced oxidative damage to DNA, proteins, and lipids. A slight response of the ROS regulatory system was apparent via increasing relative GSH levels.

In contrast, exposure of larvae to the medium Cu dose (1.10 µg·L^−1^) resulted in a rapid increase in GST and GPx activity after 3 h of exposure. After 18 h of exposure, evidence of oxidative damage to all three macromolecule biomarkers was apparent. After 42 h, DNA, protein, and lipid damage persisted with co-elevation of GR, SOD, and CAT activities. The high dose Cu treatment (50.3 µg·L^−1^) induced extensive damage to DNA, protein, and lipids in embryos after only 3 h of exposure. Unlike the sublethal Cu treatments, reduced activities of several antioxidant enzymes (i.e., GR, SOD, and CAT) were detected at the first sampling point, indicating severe toxicological effects. By 18 h, these impacts had expanded to include simultaneous reductions in GSH levels and the activities of GST and GPx.

## 4. Discussion

### 4.1. Seawater Chemistry

Most of the bulk seawater compositional features were within the range of expected values for nearshore waters in New Zealand. However, according to the Australian and New Zealand guidelines for marine water quality [21], the total Cu level measured (2.9 µg·L^−1^) was more than two-fold higher than the 95% trigger value (1.3 µg·L^−1^). Guideline trigger values are concentrations that indicate a potential environmental problem if exceeded. The percentage value is the protection level that signifies the proportion of species expected to be protected if total Cu ≤ 1.3 µg·L^−1^. Taking into consideration the concentration of dissolved organic carbon (DOC), speciation modelling predicted that the majority (ca. 95%) of the total Cu was bound to the fraction of DOC defined as humic(-like) substances. The resulting calculated level of bioavailable Cu in the bulk seawater prior to EDTA additions was 0.133 µg·L^−1^.

Speciation and competition of trace metals in aquatic environments is complex and depends on a multitude of factors, including pH, temperature, salinity, inorganic ion composition, and DOC content [34,42]. In seawater, Cu speciation is largely dominated by the formation of natural organic complexes. In the absence of a more specific characterization of the organic binding capacity of matrix, it has generally been assumed that the Cu-binding capacity of DOC is well represented by that of humic(-like) substances [45,61]. When bound to these natural organic ligands, Cu toxicity is greatly ameliorated since the free cupric ion (Cu^2+^) is largely or completely unavailable for uptake by aquatic organisms [24,62]. The remaining Cu consists of free Cu^2+^ and an array of Cu-containing inorganic compounds. In these weakly bound ionic forms, Cu is labile, bioavailable, and toxic [45].

To provide aquaculture industry-relevant information in the current study, the addition of EDTA to the incubation tanks was included to simulate commercial hatchery conditions [63]. Without EDTA, embryonic development and D-larval yield are routinely very low for this species (<10%), with occurrences of cellular membrane degradation, detachment of microvilli, perivitelline space increases, changes in behaviour, and various enzymatic and macromolecular bioindicators of severe oxidative stress [35,36,37]. Although higher survival with EDTA supplementation during embryogenesis is generally considered to be linked with the detoxification of trace metal contaminated source water, it may also be linked to the reduction of virulence factors (e.g., metal-dependent proteases) associated with ubiquitous pathogenic bacteria [64,65,66,67].

The addition of EDTA to the embryo incubation water dramatically reduced the bioavailability of Cu in the dosing treatments. The bioavailable Cu fraction (free Cu^2+^ + ∑ inorganic labile species) within each sublethal treatment was low (Table 2 [treatments 1 and 2]), with the vast majority of total Cu in these treatments being strongly bound as Cu-EDTA complexes. These findings may be of interest to the developing mussel industry in New Zealand since such levels of total Cu are beyond those that would normally be found within local coastal waters, even in highly polluted zones. A recent analysis detected total Cu levels in New Zealand mussel hatchery source water to be as high as 13.2 µg·L^−1^ [35]. This supports the continued use of metal chelators during embryo incubation as a precautionary safety measure against heavy metal contamination.

The lower treatment levels were not lethal, but they did induce dose-dependent developmental retardation, behavioural abnormalities, and structural deformities. Under the experimental conditions that were employed, data reveal that the approximate 72 h EC10 of bioavailable Cu for developmental abnormalities was around 1.10 µg·L^−1^. Comparative embryotoxicity information for the effects of Cu on *P. canaliculus* is thus far lacking; perturbation during embryogenesis may compromise subsequent larval performance, metamorphosis, and recruitment [38]. It would therefore be valuable to consider potential toxin-induced carry-over effects on later developmental stages in conjunction with sublethal embryo assessment.

### 4.2. Biochemical Profiling

Profiling of key metabolites and enzymes demonstrated that the embryonic and larval metabolomes of marine mussels are extremely sensitive to Cu-induced toxicological influences. These profiles are representative of unique ‘metabolic fingerprints’ that reflect health conditions and are thus in themselves biomarker signatures of physiological status. Distinct metabolic trajectories associated with embryonic developmental timing, levels of Cu concentration, and the duration of Cu exposure were identified. Discrimination between treatment groups was obtained via unsupervised and supervised multivariate analyses, with results showing the presence of intricate temporal metabolite variations.

One striking feature of the sublethal data was what appeared to be a sinusoidal oscillation of metabolite fold changes over time in Cu-exposed embryos compared to controls (e.g., Figure 7). This may suggest that an initial toxic shock (revealed by subtle accumulations of many metabolites in late-stage blastula embryos) was buffered via metabolic adaptation, leading to transitional returns towards baseline levels through trochophore development. However, many of these metabolites fell below control levels in early D-stage larvae, indicating potential metabolic dysregulation. This perturbation seemed to trigger an attempt to re-establish baseline metabolism during subsequent development to overcome the possible deleterious effects of sublethal treatments.

A lethal-level Cu dose caused major alterations in many metabolites and resulted in catastrophic metabolic failures and mortalities shortly after 18 h of exposure. Pathway analysis revealed changes in the levels of a number of functionally related metabolites within a range of known biochemical pathways. This approach enabled unique temporal profiles dependent on exposure duration to be discerned, which might not otherwise have been identified using more conventional procedures. As might be expected, dysregulation of these pathways became more severe with time (Figure 5). Eight out of 19 pathways showed signs of imbalance after 3 h of high Cu exposure, whereas 18 out of the 19 pathways were likely affected after 18 h of exposure. Changes in metabolite compositions indicated dysregulation of energy production, the occurrence of osmotic stress, potential neuro- and immuno-toxic associations, and numerous indicators of oxidative stress through disrupted regulation of reactive oxygen species.

#### 4.2.1. Energy Metabolism

Under normal physiological conditions, concentrations of TCA cycle intermediates remain almost constant [68]. When intermediates are removed to serve as biosynthetic precursors, they are typically replenished in dynamic balance via anaplerotic reactions (e.g., glutamine → α-ketoglutarate) [69]. However, decreases in the abundance of TCA cycle intermediates can be considered indicative of increased pathway flux, ATP demand, utilisation for secondary biosynthesis, and/or reduced mitochondrial function [70,71,72]. After 42 h of exposure, sublethal-level Cu treatments caused reductions in citrate, cis-aconitate, fumarate, and malate. These data may suggest that low concentrations of Cu alter energy metabolism in early D-stage larvae through enhanced demands for ATP to fuel detoxification processes, such as the synthesis of antioxidant enzymes. Indeed, sublethal trace metal exposures increase respiration rates in various organisms, probably to account for enhanced ATP expenditure during toxin-induced requirements for defence and repair mechanisms whilst simultaneously maintaining routine metabolism [73,74,75,76]. The decreased pool of free amino acids at this time may also indicate enrichment in protein synthesis and/or an enhanced requirement for energy through their oxidation and utilisation as TCA cycle substrates. However, these responses were transient, with levels of all detected TCA cycle intermediates returning, or adhering, to control levels by the end of the trial. This implies a capacity for metabolic regulation of immunotoxicity in developing D-larvae.

Cis-aconitate is a key substrate for the enzyme aconitase. Exposure of embryos to lethal-level Cu induced an accumulation of this metabolite, which can be attributed to reduced aconitase activity [77]. Iron-sulphur-containing aconitase is a well-known target of Cu toxicity, and, being the most sensitive TCA cycle enzyme to ROS inhibition, its activity can be used as a biomarker of oxidative stress [78,79,80,81,82]. Enzyme inhibition in the case of Cu toxicity involves O_2_˙^–^ and OH˙ produced via the Haber–Weiss reaction, a process analogous to the Fenton reaction [83,84,85]. Oxidative inactivation of aconitase promotes cluster instability, causing the enzyme to release redox-active Fe^2+^ and stimulating the associated formation of H_2_O_2_ which encourages Fenton reaction events and further production of OH˙, thus creating a self-amplifying cycle if unresolved [86,87]. Aconitase forms a direct link between ROS metabolism, iron homeostasis, the flow of metabolite intermediates in the TCA cycle, and energy production [82].

Cheng et al. [77] demonstrated via aconitase gene knockdown in a *Drosophila* model that reduced mitochondrial aconitase activity impairs glycolysis and the TCA cycle and decreases the generation of ATP. Specific markers of impaired energy metabolism caused by reduced aconitase activity included an increase in levels of cis-aconitate, and substantial concomitant decreases in downstream levels of succinate, fumarate, and malate. Results of the current study match this metabolite expression pattern for the group of embryos exposed to lethal-level Cu for 18 h, with the potential Cu/ROS-induced inhibition of aconitase limiting the synthesis and turnover of downstream TCA cycle intermediates under severe oxidative stress conditions.

#### 4.2.2. Amino Acid Metabolism

Compositional alterations in the free amino acid (FAA) pool in Cu-exposed embryos were dependent on exposure duration and level of treatment. FAAs play diverse roles in marine organisms; they represent the pool from which new proteins can be synthesised during early development, and they serve as important fuels for energy acquisition in embryos and larvae [88,89]. The FAA pool is also the receiving depot for the catabolism of endogenous protein during embryogenesis in some bivalves [90], as well as the proteinaceous components of food once feeding competency has been attained. Total free essential amino acid contents can be used as a rough proxy for protein turnover [91], and these patterns likely reflect, in part, differential capacities and/or requirements for protein production through embryogenesis and between treatments and controls. Indeed, with major perturbations to most of the FAAs detected, aminoacyl-tRNA biosynthesis being identified as a significantly affected pathway was a likely outcome due to its pivotal role in delivering amino acids to ribosomes during protein synthesis.

FAAs also have diagnostic capabilities for early detection of various pathologies and can serve as biomarkers for a number of physiological states [92]. Particular amino acids may contribute towards antioxidant protection as small peptides (e.g., glutathione) [93,94], some act as signalling molecules or precursors for their synthesis (e.g., glutamate and tyrosine) [95,96], whereas others have important biochemical roles specific for the adaptation of aquatic organisms to flourish in marine environments. For example, marine osmoconformers such as bivalve molluscs utilise certain FAAs as organic osmolytes to balance intracellular osmolality with the external medium [97].

#### 4.2.3. Osmoregulation

Free glycine, alanine, and β-alanine serve as organic osmolytes in bivalves, with sodium-dependent cotransport being responsible for their accumulation or release from tissues to maintain cell volume [97,98,99,100,101,102,103]. These osmolytes were differentially regulated in Cu-exposed embryos, compared to controls. Heavy metal toxicity appears to be strongly dependent on the abilities of some organisms to regulate intracellular osmolality. However, there are still many questions surrounding the osmoregulatory mechanistic basis for salinity-dependent Cu toxicity, especially for marine osmoconformers [102]. Nevertheless, exposure of marine bivalves to Cu and other metals induces compositional variations in FAAs with osmolytic function [103,104,105,106,107], and some new mechanistic advances have progressed our understanding of Cu-induced toxicity in these organisms. Although it has been suggested that Cu-toxicity in marine molluscs is more related to effects on acid-base equilibrium and ammonia excretion than imbalances in ion regulation, intracellular ion regulation plays a key mechanistic role [102].

Exposure of marine clam cells to Cu results in reductions of intracellular Na^+^, K^+^, and Cl^−^ concentrations, which are presumed to be associated with inhibition of Na^+^/K^+^-ATPase activity and competition by Cu for cell membrane ion transport systems, such as Na^+^ channels and the Na^+^/K^+^/2Cl^−^ cotransporter [108,109]. However, although haemolymph osmolality and levels of Na^+^ and K^+^ are not altered by Cu exposure in clams, Na^+^/K^+^-ATPase expression increases in gills and digestive glands [110,111]. This suggests that increased Na^+^/K^+^-ATPase expression may be a compensatory physiological response to ameliorate the metal-induced inhibition of enzyme activity. Additional Cu-induced changes on intracellular levels of divalent cations (Mg^2+^ and Ca^2+^) have also been observed in clams [111], and Ca^2+^ flux has been linked with organic osmolyte transport systems in mussels and clams [112,113]. Thus, it is becoming clearer that Cu is an ionoregulatory toxicant in marine bivalves, affecting ionic and volume control at the cellular level.

#### 4.2.4. Neurotoxicity

Neurotoxicity may have been an important mechanistic feature contributing to deteriorating health in Cu-exposed embryos. We recently established that neurogenesis in *P. canaliculus* commences early on during development, with expressions of solitary FMRF-amidergic cells being identifiable via immunochemical labelling at the trochophore stage (18 hpf) [39]. By the early D-larval stage (42 hpf), immunoreactivity patterns within neural compartments are consistent with the initial formation of the cerebral ganglion, which quickly progresses to include the emergence of the peripheral system and mantle nerve within a couple of days. In the current study, levels of a number of amino acids associated with neurotransmission were found to be differentially affected. Pathway enrichment analysis also substantiated this with the detection of significant network alterations to glutamine and glutamate metabolism, as well as phenylalanine, tyrosine, and tryptophan metabolism.

Phenylalanine and tyrosine are precursors for the biosynthesis of L-DOPA and the catecholamine signalling messengers, dopamine and epinephrine. These amino acids and/or their neuroactive derivatives can modulate larval behaviour in different mussel species, including *P. canaliculus* [40,114,115]. Such metabolites also have the ability to regulate larval swimming activities and developmental timing of other molluscan taxa through their actions on ciliary beat frequencies of innervated velar tissues and morphogenetic programmes, respectively [116]. Phenylalanine and tyrosine are tightly coupled with the production of endogenous catecholamines in molluscs and are thought to regulate neuronal processes in developing embryos and larvae. For example, enhancement of tyrosine or L-DOPA concentrations in molluscan tissues leads to increased catecholamine synthesis [117,118,119], and inhibition of the catecholamine re-uptake system in oyster embryos leads to developmental retardation, a reduction in growth rate, and the formation of shell structure abnormalities in early D-stage larvae [120].

Neurotoxic effects of heavy metals on metabolite intermediates associated with catecholamine biosynthesis have been described in various model invertebrates. Exposure of nematodes (*Caenorhabditis elegans*) to sublethal lead levels reduces whole animal tissue concentrations of free phenylalanine and tyrosine and is suggested to contribute towards toxicity through deleterious neuronal consequences of atypically enhanced catecholamine production [121]. Short-term exposure of water fleas (*Daphnia magna*) to sublethal levels of metals (Cu, Li, and/or Cd) for 24–48 h also causes a reduction in phenylalanine content, increases tyrosine hydroxylase gene expression, and enhances production of L-DOPA and dopamine [122,123]. These coordinated precursor-product alterations are highly supportive of a mechanistic link, and the upregulation of this pathway may be a general response to metal stress [123].

Tryptophan is the precursor of serotonin, which plays an important role during early embryogenesis in molluscs [124,125,126,127]. Serotonin is involved in oocyte maturation, fertilisation, and cleavage divisions, and is associated with embryonic/larval sensory organs, the regulation of embryonic rotations, and controlling development rates and larval behaviours [127,128,129]. Studies on Cu-induced neurotoxicity via modulation of tryptophan metabolism and disruption of the serotonergic nervous system are currently lacking in molluscan models. However, mechanistic insights from fish studies, e.g., [130,131,132,133,134], may provide foundations for hypothesis generation.

Glutamine is the common precursor for the biosynthesis of glutamate and ƴ-aminobutyric acid (GABA). Glutamate is considered a major excitatory neurotransmitter, whereas GABA is a major inhibitory neurotransmitter across vertebrate and invertebrate taxa. However, in particular, molluscan neurons can act in both excitatory and inhibitory modes [135]. When applied exogenously to seawater, these metabolites modulate larval swimming and attachment behaviours in molluscs and induce fast synchronous metamorphosis in sea urchin larvae [116,136,137,138,139]. Glutamatergic neurons are also identifiable in the peripheral and central nervous systems of ascidian and molluscan larvae, with presumed functions in mechanical and sensory processes [140]. Together, these observations signify various neuroactive roles during early marine invertebrate development.

Glutamine and glutamate have been suggested as useful biomarkers of neurotoxicity in adult mussels exposed to heavy metal contamination [103,141]. Differential regulation of glutamine and glutamate metabolism as a neurotoxic response to Hg, Zn, and Cd has also been demonstrated in fish and clams [142,143,144], and Cd and/or Cu exposure influences glutamine synthetase mRNA expression and/or glutamate dehydrogenase activity in mussels and fish [145,146]. Excessive levels of Mn, Hg, and Pb trigger neurotoxicity through similar disruptions of the glutamine/glutamate-GABA cycle, which underlines a common mechanism of metal toxicity [147,148]. Wirbisky et al. [149] demonstrated that Pb exerts neurotoxicity in fish embryos by influencing the GABAergic system in a dose-responsive and temporally variable manner through embryogenesis. Our results indicate that glutamine and glutamate biosynthesis in mussel embryos are similarly perturbed by Cu exposure, which is consistent with the findings of these studies and further suggests that this system may also be a useful biomarker of metal-induced neurotoxicity in molluscan embryos.

#### 4.2.5. Pyrimidine Catabolism

The pyrimidine nucleotides thymine and cytosine are catabolised in mussels to 3-aminoisobutyric acid (BAIBA) and β-alanine through their uracil derivatives, respectively [150]. Both degradation products were found to be higher in mussel embryos exposed to lethal-level Cu. Since β-alanine serves osmoregulatory functions in marine bivalves associated with Cu-induced toxicity and sodium-dependent co-transport, tissue concentrations are likely to reflect the net outcome of the interplay between these systems. BAIBA can also be used as an approximate indicator for the rate of DNA and tRNA turnover [151]. Increases in BAIBA have been found in different animal models exposed to lead and insecticides, with toxin-induced DNA/RNA damage thought to be responsible for the elevated levels observed [152,153,154,155]. The higher levels of pyrimidine degradation products measured in our study are indicative of shifts in nucleotide metabolism, with potential adverse implications for DNA/RNA production, protein synthesis, and organismal growth. Links between pyrimidine metabolism and oxidative stress have been implicated in a number of disease pathologies and metal toxicity responses [156,157,158,159,160,161,162].

#### 4.2.6. Oxidative Stress, Redox Homeostasis, and Metal Chelation

The generation of reactive oxygen species (ROS) and oxidative stress is one of the most well-known mechanisms of metal toxicity in aquatic organisms [163,164]. A number of metabolite signatures associated with oxidative stress responses and/or with roles in the maintenance of redox homeostasis or metal chelation were identified. These included increased levels of ROS, changes in aminomalonic acid levels, variation in histidine metabolism, alterations to the transsulfuration pathway, differential regulation of glutathione (GSH) metabolism, and the presence of lipid peroxidation by-products. The metabolite signatures of an oxidative stress response were further verified via targeted analyses of enzymes involved in ROS regulation and macromolecular oxidative damage to DNA, proteins, and lipids.

Aminomalonic acid (AMA) showed variable flux in embryos exposed to Cu. When protein-/peptide-bound, AMA has possible origins stemming from errors in protein synthesis and oxidative damage to amino acids [165]. The moiety is generated by free radical oxidation of proteins and can arise due to hydrogen atom abstraction from the α-position of glycine residues [166]. The dicarboxylic acid can also be derived from metal-induced cysteine oxidation via β-elimination of the sulphur residue [167]. Reductions in free AMA within rabbit hepatic tumours are suspected due to depletion of cysteine stemming from enhanced requirements for GSH to alleviate oxidative stress [168], and toxin-induced changes in the free metabolite have previously been recorded in water fleas exposed to polyaromatic hydrocarbons [169].

Free histidine can serve as a high affinity chelator of metals, and it has been suggested that elevation of histidine levels as a response to Cu toxicity may provide an energetically low-cost detoxification mechanism for invertebrates [170], as is the case for plants [171,172]. However, Bundy et al. [173] comprehensively tested the hypothesis that Cu exposure upregulates histidine metabolism in earthworms and found a weak negative correlation between levels of free histidine and Cu dose. Digilio et al. [174] conducted a metabolomics-based investigation of Cu toxicity in adult mussels and found a parallel trend of subtle histidine reductions in haemolymph samples with increasing doses of Cu. Exposure of mussel embryos to lethal-level Cu in the present study similarly caused histidine levels to decrease in mussel embryos. Incorporation of histidine into histidine-rich glycoprotein (HRG) may be acting as a detoxification strategy against excess metal concentrations. HRGs are important immunoresponsive macromolecules that are widely distributed across bivalve taxa and have very high capacities to bind and detoxify divalent metal ions such as Cu [175,176].

The transsulfuration pathway involves the conversion of methionine to cysteine, via cystathionine, and is strongly affected by metal exposure [177,178,179,180]. Cysteine and methionine metabolism were differentially regulated in mussel embryos exposed to Cu, with relative metabolite levels involved indicating depletion of methionine through enrichment in transsulfuration, and subsequent downstream metabolism of cysteine. Cysteine is an important structural component of glutathione (GSH), and the rate-limiting precursor in its biosynthesis [181]. When the demand for GSH is high due to generation of ROS, the transsulfuration pathway can act as a reserve pathway that channels methionine towards cysteine (through initial conversion to homocysteine, subsequent conjugation with serine to form cystathionine, and then final cleavage and transsulfuration of the carbon-sulphur bond) [182]. In its free form, cysteine’s thiol group gives it an effective capacity to chelate metal ions. However, when bound to redox-active metals, such as copper, free cysteine is quickly oxidised, and the reduced metal may undergo a Fenton-like reaction to form highly toxic hydroxyl radicals (˙HO); thus, keeping free cysteine levels low is necessary to protect against these harmful oxidants [183]. Conversely, as a residue within GSH, cysteine’s amino group is blocked via conjugation with glutamate, and thiol oxidation by transition metals is greatly diminished so as to prevent deleterious Fenton-like reactions from occurring, while simultaneously providing a tripeptide with strong antioxidant properties [183]. Jeppe et al. [179] reported that exposure of aquatic midge larvae to Cu resulted in differential expression patterns of transsulfuration genes, advocating that Cu induces an increased flux of cysteine into GSH synthesis, while, at the same time, diminishing pools of cystathionine. Our results appear to be consistent with these findings.

Lethal-level Cu treatment caused free methionine, cystathionine, and cysteine to decrease markedly in mussel embryos, which may have been a metabolic attempt to alleviate oxidative stress caused by the metal. Such responses could prevent Fenton reactions from occurring and also be indicative of an enhanced requirement for GSH production and antioxidant activity. It appears that Cu, Cd, and Zn may have quite diverse mechanisms of toxicity regarding their particular impacts on transsulfuration pathway components [179,180]. For example, Cu and Cd differentially modulate transsulfuration pathway metabolites, suggesting that different pathway components are being targeted by the toxins [179]. Although many transition metals share some mechanistic similarities in their toxic actions, it has been suggested that the transsulfuration pathway could be used as a specific diagnostic biomarker suite for detecting the identity of distinct metal stressors [180].

Pathway enrichment and topology analysis identified GSH metabolism as being differentially regulated after lethal-level Cu exposure. The network includes the metabolites cysteine, glycine, glutamic acid, GSH, ornithine, and generic collectively termed L-amino acids, all of which were detected by our methods. Copper exposure caused levels of GSH to fall, which was an expected response to Cu toxicity since metals increase GSH oxidation, resulting in a depletion of cellular GSH under high oxidative stress conditions [184]. GSH is an efficient antioxidant and provides protection against oxidative stress by conjugating with electrophiles and reducing ROS [184]. During this process, GSH is converted into its oxidised form (GSSG) via catalysis by GPx. GSH is also involved in the formation of GSH-S conjugates with ionic forms of Cu (forming linear II covalent complexes) and acts as an intracellular chelator to prevent the nucleophilic interaction of Cu with the main cellular structures [185,186]. Lower-level Cu exposures in mussel embryos caused transient responses in GSH pathway metabolites, which were indicative of variations between oxidative stress-induced GSH demand, biosynthesis, and oxidation. Under low-to-moderate oxidative stress conditions, GSH concentrations may be replenished through biosynthesis from precursor metabolites and/or through the NADPH-dependent reduction of GSSG [187,188]. Indeed, the maintenance of basal-level GSH flux during sublethal Cu treatments is indicative of a well-functioning GSH homeostatic control mechanism.

ROS homeostasis and GSH turnover are regulated by an elaborate system of enzymes, antioxidants, and pro-oxidants. Cu-induced toxicity responses of different biochemical components accountable for controlling redox balance in aquatic organisms can vary widely depending on the duration of Cu exposure, the Cu concentration, the type of tissue assessed, the cellular localisation of the biomarkers themselves, and also the particular enzyme isoform(s) being considered [189,190,191,192,193]. The response patterns of ROS and activity levels of enzymes involved in ROS regulation and GSH turnover provide clear complementary evidence that oxidative stress was occurring in Cu-exposed embryos. This was further verified by levels of oxidative damage to DNA, proteins, and lipids in developing embryos.

#### 4.2.7. Lipid Metabolism

Prior to attaining feeding competency, lipids are the most important sources of energy during embryonic and larval development for many bivalve species [194,195,196]. When energy requirements are high, lipases hydrolyse triacylglycerols and release fatty acids (FAs). These free fatty acids (FFAs) are rapidly metabolised to acetate by β-oxidation in the mitochondria and ultimately enter the TCA cycle under optimal conditions to supply ATP for various essential cellular processes (anabolic reactions, active transport, cell division, motility, muscular contraction, etc.). Increased lipid catabolism, and/or enriched FA biosynthesis, and/or reduced FFA consumption can all lead to apparent rises in the total FFA pool, and vice versa.

Bivalve oocytes and embryos contain high levels of maternally derived FA desaturase mRNA for altering the degree of FA saturation during early development and can therefore perform certain fatty acid conversion tasks (e.g., C20:4[n-3] to C20:5[n-3]) [197,198]. However, they typically do not have appreciable levels of elongase activity prior to attaining feeding competency and are therefore unlikely to be able to generate fatty acids from acetyl-CoA subunits, or from smaller fatty acid precursors [199]. Thus, variations in levels of FFAs in the current study are more likely indicative of altered lipid metabolism than changes in de novo FFA biosynthesis.

With increases in FFA pools and greater demands for energy under enhanced oxidative stress conditions, it seems reasonable to suggest that lipid catabolism was being upregulated in certain Cu-treated groups. At the same time, reduced TCA cycle flux due to extreme Cu toxicity on enzyme activities may have simultaneously blocked FFA consumption, leading to extreme FFA accumulations, as was indicated under lethal-level Cu exposures by the relative levels of TCA cycle intermediates.

#### 4.2.8. Immunotoxic Associations

FFA accumulations are associated with mechanisms of immunotoxicity. Increases in the FFA pool can induce oxidative stress through ROS generation and stimulate inflammation via upregulation of the redox-sensitive transcription factor Nuclear Factor-[kappa]B (NF-κB) [200,201,202]. These processes are not exclusive of one another. FFA-induced oxidative stress and harmful proinflammatory responses are inextricably linked via crosstalk between ROS and the NF-κB pathway. For example, while ROS have various inhibitory or stimulatory roles in NF-κB signalling, certain NF-κB-regulated genes serve major functions in mediating cellular ROS levels [203]. The NF-κB pathway is crucially involved in many biological processes, including innate immune responses in molluscs, inflammation, cell proliferation and differentiation, apoptosis, and embryonic morphogenesis [204,205,206,207,208,209,210]. In addition, it has been established that Cu-induced immunotoxicity involves activation of the NF-κB pathway via direct or indirect ROS-mediated effects on the transcription factor [211,212]. Thus, the large increases in the total FFA pool that were observed in moribund embryos exposed to lethal-level Cu both indicate and promote a high level of oxidative stress and inflammation. This consequence was not apparent from the metabolomics data after short-term (3 h) lethal-level exposure or significant at any time during sublethal treatments. This may suggest that the response occurs due to severe failures of the system only under high Cu dose conditions. Under sublethal conditions, FFA consumption and conversion through a less negatively impacted TCA cycle to deliver the required energy demands may have been responsible for circumnavigating the potentially damaging immunotoxic effects of excessive FFA accumulations.

#### 4.2.9. Other Metabolic Responses

Malonic acid levels were enhanced by lethal-level Cu exposure. Elevated malonic acid was recently reported in shrimp and oysters from sites contaminated by industrial discharges with high concentrations of trace metals [213,214,215]. Malonic acid is a C3 dicarboxylic acid and is well-known for its regulatory role in cellular respiration by competing for the active site of succinate dehydrogenase/respiratory complex II (SDH/CII), which catalyses the oxidation of succinate to fumarate in the TCA cycle [216,217]. Enzyme inhibition would reduce the amount of fumarate being synthesised, which is in accordance with the diminished levels of fumarate that were detected in the Cu-exposed embryos. The induced mitochondrial dysfunction caused by SDH/CII inhibition can, in turn, trigger ROS production, which depletes GSH and NADPH, thus overwhelming mitochondrial antioxidant capacity and resulting in lipid peroxidation and mitochondrial swelling [218]. Malonic acid is also associated with osmotic adjustment and secondary neuronal excitotoxicity and can potentiate the release of cytochrome c to induce apoptosis [218,219].

The C5 dicarboxylic acid glutaric acid is an endogenous toxin also known to induce oxidative stress [220]. Lethal-level Cu caused levels of glutaric acid to increase in mussel embryos, whereas sublethal exposures did not. Although the mechanisms involved are not yet fully characterised, increased metabolite levels have been associated with decreased GSH concentrations, reduced GSH peroxidase activity, depressed total antioxidant reactivity, and increased lipid peroxidation and protein carbonylation [221,222]. Glutaric acid also plays roles in apoptotic pathways and can impair energy metabolism through inhibition of Na^+^/K^+^-ATPase and enzymes involved in oxidative phosphorylation [221,223].

2-Aminobutyric acid (2-ABA) has also been linked with mechanisms of oxidative stress [224]. Under cysteine deficiency caused by considerable oxidative stress and high GSH demand, the first enzyme (glutamate cysteine ligase) of the GSH biosynthetic pathway utilises 2-ABA instead of cysteine (due to its similar structure) and causes the production of ophthalmic acid instead of GSH [225,226]. Thus, reductions in 2-ABA provide additional evidence within the metabolomics data for severe dysregulation of the redox system under conditions of high Cu contamination. This effect was not observed after sub-lethal Cu exposures, with ROS homeostasis and GSH demand seemingly being maintained within certain acceptable boundaries.

### 4.3. Metabolomics as a Health Assessment Tool

The capacity of metabolomics to discriminate larval groups in this study was far more powerful than using routine visual assessment methods. The lowest sublethal-level copper exposure caused some (~10%) changes in D-larval swimming behaviour (i.e., slowed swimming and altered motion trajectories) towards the end of the trial, but did not impact the rate of embryonic development. Metabolomic analysis of these samples identified significant compositional changes in embryonic metabolite profiles after only 3 h of exposure, and similarly could discriminate treated embryos/larvae at every subsequent sampling point from non-treated controls. This highlights the sensitivity that metabolomics can offer to detect changes in metabolic processes days before organisms display any visually observable phenotypic traits of an altered health state. These findings also suggest that metabolomic approaches can be used for the early detection of poor larval health and have potential capacities for prognosis and diagnosis.

Within the context of molluscan aquaculture, there is positive scope for the application of metabolomics to monitor larval health during hatchery culture. The causes of poor larval health and incidences of mass mortality within hatcheries are often undefined; being able to identify the precise timing of physiological ‘tipping points’ could inform a re-evaluation of husbandry practices. Metabolite profiling is also clearly useful for characterising mechanisms of toxicity and could be incorporated into ecotoxicological test methods to provide information earlier than traditional biological endpoints, such as ‘% D-larval yield’ and mortality. With further development and biomarker validation, metabolomic approaches could provide additional measures of toxicity, enhance test sensitivity, and reduce test duration.

## 5. Conclusions

Simultaneous profiling of metabolites, enzymatic biomarkers associated with the maintenance of ROS homeostasis, and non-enzymatic biomarkers associated with the oxidative damage to key cellular components clearly demonstrated the occurrence of oxidative stress in mussel embryos as a response to Cu. Untargeted and targeted analytical strategies can be used effectively to gain highly complementary information. Metabolomic analysis was more sensitive at discriminating copper-exposed embryos/larvae from non-exposed organisms compared to analysis of oxidative stress biomarkers since the lowest Cu dose altered the embryonic metabolome but not the ROS regulatory system. This indicates that metabolic processes can be affected by Cu as a first consequence in the absence of an oxidative stress mechanism. Furthermore, it appears that metabolic regulation of low-level Cu toxicity can be achieved effectively without significant inputs from the primary redox balance system. Metabolomics is a useful approach to characterising oxidative stress and revealing additional insights into mechanisms associated with metabolic dysfunctions that underpin poor embryonic/larval health in marine invertebrates.

## Figures and Tables

**Table 1:** Bulk seawater composition prior to EDTA and CuSO_4_ additions.

Copper Species/Fraction	Concentration	Element	Concentration	Element	Concentration
Total dissolved Cu	2.90 µg·L^−1^	Aluminium	<13.0 µg·L^−1^	Magnesium	1.49 g·L^−1^
Humic-bound Cu	2.76 µg·L^−1^	Arsenic	<4.2 µg·L^−1^	Manganese	<1.1 µg·L^−1^
Free Cu^2+^	0.01 µg·L^−1^	Barium	5.9 µg·L^−1^	Mercury	<80.0 ng·L^−1^
Inorganic-bound Cu	0.123 µg·L^−1^	Beryllium	<0.63 µg·L^−1^	Molybdenum	11.1 µg·L^−1^
Bioavailable Cu	0.133 µg·L^−1^	Boron	4.0 mg·L^−1^	Nickel	<6.3 µg·L^−1^
**Nutrient Profile**	**Concentration**	Bromine	82.0 mg·L^−1^	Phosphorus	22.0 µg·L^−1^
Total ammoniacal-N	<10.0 µg·L^−1^	Cadmium	<0.21 µg·L^−1^	Potassium	0.4 g·L^−1^
Nitrite-N	<2.0 µg·L^−1^	Cesium	<1.9 µg·L^−1^	Rubidium	0.12 mg·L^−1^
Nitrate-N	4.0 µg·L^−1^	Calcium	0.42 g·L^−1^	Selenium	<4.2 µg·L^−1^
Nitrite-N + Nitrate-N	4.0 µg·L^−1^	Carbon	24.0 mg·L^−1^	Silver	<0.43 µg·L^−1^
Dissolved reactive P	8.0 µg·L^−1^	Chloride	19.0 g·L^−1^	Sodium	11.2 g·L^−1^
**Other**	**Concentration**	Chromium	<1.1 µg·L^−1^	Strontium	8.7 mg·L^−1^
Alkalinity (CaCO_3_)	116.0 mg·L^−1^	Cobalt	<0.63 µg·L^−1^	Thallium	<0.21 µg·L^−1^
Total Kjeldahl Nitrogen	<0.2 mg·L^−1^	Fluoride	1.6 mg·L^−1^	Tin	<1.7 µg·L^−1^
Reactive silica (SiO_2_)	0.29 mg·L^−1^	Iron	5.4 µg·L^−1^	Uranium	3.3 µg·L^−1^
Dissolved non-purgeable organic C	1.1 mg·L^−1^	Lead	<1.1 µg·L^−1^	Vanadium	1.9 µg·L^−1^
		Lithium	0.19 mg·L^−1^	Zinc	6.3 µg·L^−1^

**Table 2:** Between-treatment variation in different copper fractions within incubation tanks after EDTA addition and three levels of CuSO_4_ treatment. Note differences in concentration units.

Copper Species/Fraction	Experimental Copper Concentrations			
	Control	Low Dose	Medium Dose	High Dose
Target total dissolved Cu	0.0 µg·L^−1^	100 µg·L^−1^	200 µg·L^−1^	300 µg·L^−1^
Measured total dissolved Cu	2.6 µg·L^−1^	130 µg·L^−1^	250 µg·L^−1^	370 µg·L^−1^
Humic-bound Cu	26.0 ng·L^−1^	1.6 µg·L^−1^	9.0 µg·L^−1^	57.0 µg·L^−1^
EDTA-bound Cu	2.6 µg·L^−1^	130 µg·L^−1^	240 µg·L^−1^	260 µg·L^−1^
Free Cu^2+^	0.06 ng·L^−1^	3.2 ng·L^−1^	83.0 ng·L^−1^	4.3 µg·L^−1^
Inorganic-bound Cu	4.0 ng·L^−1^	39.0 ng·L^−1^	0.98 µg·L^−1^	51.0 µg·L^−1^
Non-bioavailable Cu	2.6 µg·L^−1^	130 µg·L^−1^	249 µg·L^−1^	310 µg·L^−1^
Bioavailable Cu	0.47 ng·L^−1^	0.04 µg·L^−1^	1.1 µg·L^−1^	50.3 µg·L^−1^

**Table 3:** Relative temporal changes in oxidative stress biomarkers across Cu treatments compared to controls. Up/down-arrows represent statistical differences (ANOVA; Dunnet’s post hoc tests; *p* < 0.05) between treatments and controls displayed in Figure 9 at different time points; hyphens represent non-statistical differences.

Biomarker	0.04 µg·L^−1^				1.10 µg·L^−1^				50.3 µg·L^−1^	
	3 h	18 h	42 h	72 h	3 h	18 h	42 h	72 h	3 h	18 h
DNA damage	-	-	-	-	-	↑	↑	↑	↑	↑
Protein damage	-	-	-	-	-	↑	↑	↑	↑	↑
Lipid damage	↓	-	↑	-	-	↑	↑	↑	↑	↑
GSH	-	-	-	-	-	-	-	-	↓	↓
CAT	-	-	-	-	-	-	↑	↑	↓	↓
SOD	-	-	-	-	-	-	↑	↑	↓	↓
GR	-	-	-	-	-	-	↑	↑	↓	↓
GST	-	-	-	-	↑	-	↑	↑	-	↓
GPx	-	-	-	-	↑	↑	↑	↑	-	↓

## Data Availability

The datasets generated during the current study are not publicly available due to commercial sensitivity but are available from the corresponding author on reasonable request.
